# Thermoelectric Cement Composite Materials for Energy Harvesting and Self-Powered Sensing

**DOI:** 10.34133/research.1454

**Published:** 2026-10-27

**Authors:** Chen Zhang, Yinghui Wu, Menghuan Guo, Yingwu Zhou, Kang Jiang, Feng Xing

**Affiliations:** ^1^ Guangdong Provincial Key Laboratory of Durability for Marine Civil Engineering, College of Civil and Transportation Engineering, Shenzhen University, Shenzhen, China.; ^2^ Key Laboratory of Marine Civil Engineering Materials and Structures (Ministry of Education), School of Mechanics and Construction Engineering, Jinan University, Guangzhou, China.

## Abstract

The share of building energy consumption in global total energy consumption continues to rise. Transforming building envelopes into media for low-grade heat harvesting and self-powered sensing has therefore become one of the key technological pathways toward achieving the “dual carbon” goals. In recent years, ionic thermoelectric (i-TE) materials have attracted widespread attention due to their operational capability under low temperature gradients, readily available raw materials, and high thermoelectric potential. Endowing commodity building materials with i-TE functionality offers an effective strategy for low-grade waste heat harvesting in buildings and their low-carbon transition. Cement-based materials, owing to their natural porous structure, abundant pore solution ions, and multiphase solid–liquid interfacial environment, exhibit unique potential as i-TE media. This paper systematically reviews the research progress on the i-TE performance of i-TE cement-based composites (i-TECs). The core mechanisms of the thermodiffusion effect and thermogalvanic effect are explained, and a framework for evaluating key performance parameters is established. The paper examines how the intrinsic properties of the cement matrix govern i-TE behavior and summarizes the strategies currently employed to optimize performance. The applications of i-TECs in building energy harvesting, self-powered temperature sensing, and structural supercapacitor energy storage are surveyed, highlighting the multifunctional potential of these materials. Finally, this review outlines the key challenges in guiding the rational development of durable and efficient i-TECs, and provides an outlook on the development prospects and engineering application potential.

## Introduction

Rising global energy demand, along with the environmental and climate impacts of excessive fossil fuel consumption, is accelerating the transition toward sustainable energy systems [1]. Nowadays, building-related energy consumption is accounting for up to 40% of global energy consumption [2]. Therefore, reducing their operational energy use and even enabling them to actively generate energy has become a key step toward carbon peaking and carbon neutrality [3,4]. Building envelopes (e.g., walls, roofs, and pavements) are continuously exposed to the environment and directly receive heat flows from solar radiation, ground heat conduction, and indoor–outdoor temperature differences (∆*T*) [5,6]. If this widely available low-grade heat (<100 °C) could be harvested effectively, it would offer a distributed, self-sustaining power supply for wireless sensors, environmental monitors, and Internet of Things (IoT) devices inside buildings. This would provide new solutions for energy-autonomous buildings and smart infrastructure.

Thermoelectric (TE) materials can directly convert heat into electricity through the Seebeck effect. They offer the advantages of no vibration and wear, silent operation, and zero direct emissions and have therefore attracted widespread attention [7,8]. By incorporating TE functional fillers into cement, researchers have developed TE cement-based composites (TECs) that combine structural load bearing with energy harvesting, thereby opening a new route toward sustainable infrastructure [9,10]. Carbon-based fillers (such as carbon fibers (CFs) [11,12], carbon nanotubes (CNT) [13,14], expanded graphite (EG) [15,16], and graphene (GP) [17,18]) have been introduced into the electrically insulating cement matrix to build conductive networks that enhance carrier transport [19,20]. Firstly, researchers observed a TE effect in CF-reinforced cement, reporting a Seebeck coefficient (*S*) approaching 0.018 mV K^−1^ [11]. Metal compounds [21,22] and their oxides (e.g., MnO_2_ [23], ZnO [24], Fe_2_O_3_ [25,26], and Ca_3_Co_4_O_9_ [27]) were also shown to substantially improve the TE properties of cement-based materials. Building on these findings, TECs have demonstrated considerable promise for urban building heat harvesting [28–31], structural health monitoring [32–34], road de-icing [35,36], and self-powered cathodic protection of structures [37,38]. Despite this progress, conventional electronic thermoelectric (e-TE) materials (such as inorganic semiconductors like Bi_2_Te_3_ and SnSe) typically face bottlenecks that include scarce raw materials, high processing costs, and intrinsic brittleness [39]. In addition, carbon-based fillers require complex alignment or dispersion processes. As a result, the power densities achievable from TECs remain low, making them difficult to meet the demands of large-area distributed energy harvesting in buildings.

Ionic thermoelectric (i-TE) materials provide an effective solution, as their thermal voltage is far higher than those of e-TE. Unlike the Seebeck effect, which relies on electron/hole diffusion, i-TE materials use ions as charge carriers and generate thermal voltage through the Soret effect or the thermogalvanic effect [40,41]. Their key advantage lies in their ability to achieve ionic Seebeck coefficient (*S_i_*) on the order of mV K^−1^, whereas e-TE materials typically show only tens to 10^1^ to 10^2^ μV K^−1^ [42]. Capitalizing on this high TE potential, introducing ionic conductive materials into cement matrix can produce composite systems with an i-TE response, thereby making it feasible to harvest low-grade environmental heat from buildings [43,44].

Cement-based materials are not only the most widely used building materials, but their inherent properties also provide an ideal platform for inducing and regulating ion thermal diffusion. Their abundant pore structure (ranging from gel pores of 2 to 10 nm to capillary pores of 10 to 100 μm) provides natural ion transport channels [45]. The pore solution is rich in OH^−^, Na^+^, K^+^, and Ca^2+^, and pH is typically maintained between 12.4 and 13.5 [46], providing favorable conditions for ion transport driven by ∆*T*. Preliminary efforts have demonstrated that ions in ordinary Portland cement (OPC) can generate thermal voltages under a temperature gradient [47–49], but research on the intrinsic i-TE effect of i-TE cement-based composites (i-TECs) remains in its infancy. It has been reported that the inherently low ionic conductivity (*σ_i_*) of cementitious systems (typically 10^−3^ to 10^−1^ S m^−1^) increases the difficulty of achieving high output power in i-TECs [50]. Meanwhile, experimental thermal voltages reported for OPC vary widely, ranging from a few microvolts to several millivolts [51–53]. This large scatter arises because cement-based materials are complex, multiscale, multiphase systems in which porosity, moisture content, degree of hydration, and the ionic composition of the pore solution all exert coupled effects on the *S_i_* [49]. At present, researchers have sought to systematically enhance i-TE performance by developing various types of cement, introducing functional fillers and optimizing the pore structure. For example, a hierarchically structured poly(vinyl alcohol) (PVA) hydrogel–cement composite achieved an *S_i_* of −40.5 mV K^−1^ and a figure of merit (*ZT*) of 6.6 × 10^−2^ [54], representing the best i-TE performance reported to date. Nevertheless, there is still a lack of systematic understanding of how the cement microstructure and external conditions influence i-TE output, and the improvement strategies and application requirements of cement composite materials.

In view of this, this paper provides a comprehensive overview of recent progress on i-TECs, covering their matrix, principles, devices, and applications (Fig. 1A). First, the microscopic physical mechanisms of the i-TE effect are described, along with the factors that influence key performance parameters and the strategies for controlling them. The synergistic influence of pore structure, hydration products, and ionic species on i-TE performance is then thoroughly analyzed. Next, the current optimization approaches for enhancing the *S_i_* and *σ_i_* of i-TECs are systematically summarized. Energy storage applications based on i-TECs and their prospects in structural health monitoring are further discussed. Finally, the key challenges in this field are highlighted, and an outlook on future directions and potential applications is provided, aiming to provide theoretical support and technical references for the performance optimization and engineering deployment of i-TECs.

**Fig. 1.**
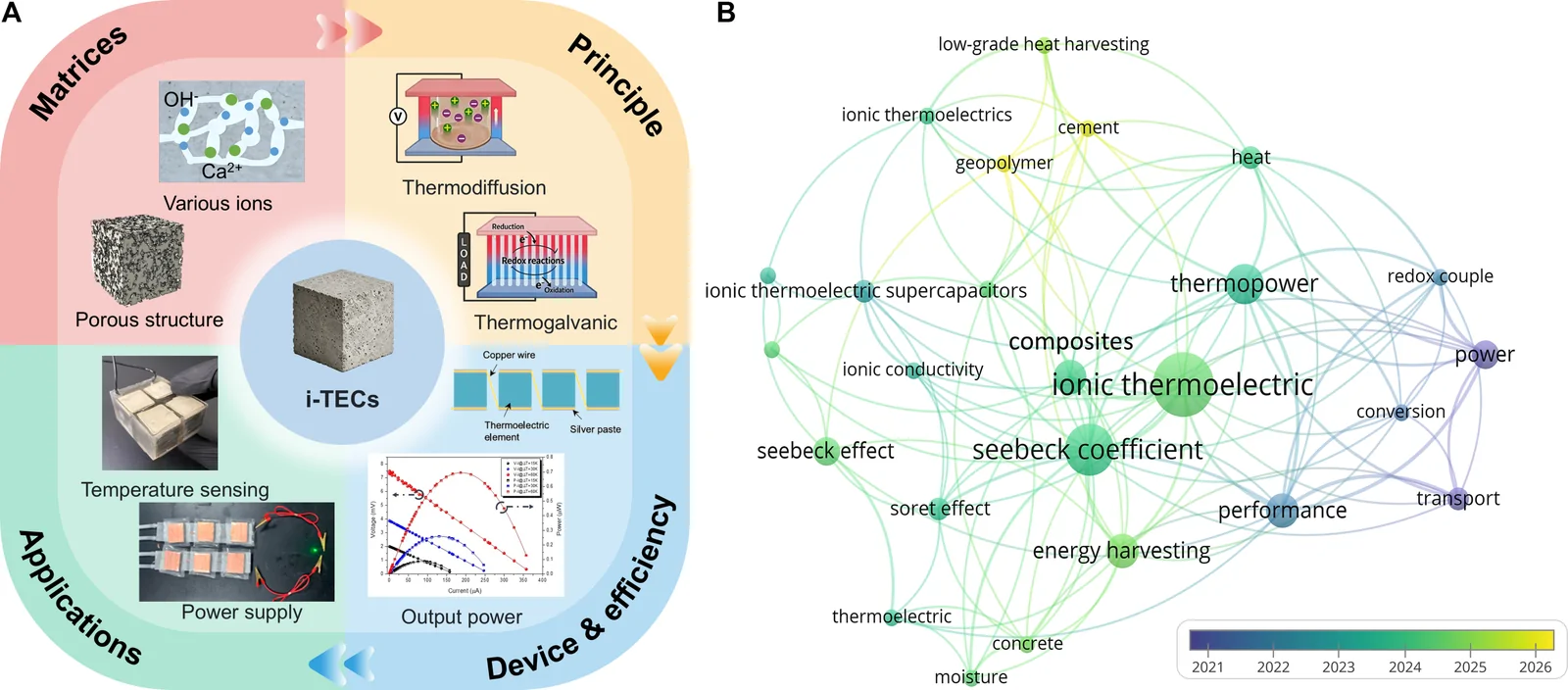
(A) Schematic illustration of i-TECs, including matrices, principle, devices, and applications. (B) Key words and their correlations of papers on i-TECs from 2021 to 2026. Data were retrieved from the Web of Science Core Collection database. Reproduced with permission [48]. Copyright 2023, Elsevier [158]. Copyright 2024, Royal Society of Chemistry. Reproduced with permission [143]. Copyright 2024, Elsevier. Reproduced with permission [146]. Copyright 2026, American Chemical Society.

This review was conducted following a systematic literature search strategy. Preferred reporting items for systematic reviews and meta-analyses (PRISMA) flow diagram is used for the literature identification, screening, eligibility, and inclusion process. Based on the results, the keywords of the relevant papers over the past 5 years were plotted using the VOS viewer software, as shown in Fig. 1B.

## Theory and Characterizations of i-TE Effect

### i-TE mechanisms

Both e-TE and i-TE materials can convert thermal energy into electrical energy, but their mechanisms differ. The e-TE effect (Seebeck effect) refers to the phenomenon where charge carriers (electrons or holes) generate a thermal voltage inside a sample when a ∆*T* exists across its 2 ends [55,56], as shown in Fig. 2A(i). Unlike e-TE materials, i-TE effect refers to the phenomenon where a potential difference is formed due to the difference in thermal diffusion rates of positive and negative ions driven by a temperature gradient within a sample. The generation of its i-TE potential can be attributed to 2 core mechanisms: the thermodiffusion effect [57] and the thermogalvanic effect [58] [Fig. 2A(ii) and (iii)].

**Fig. 2.**
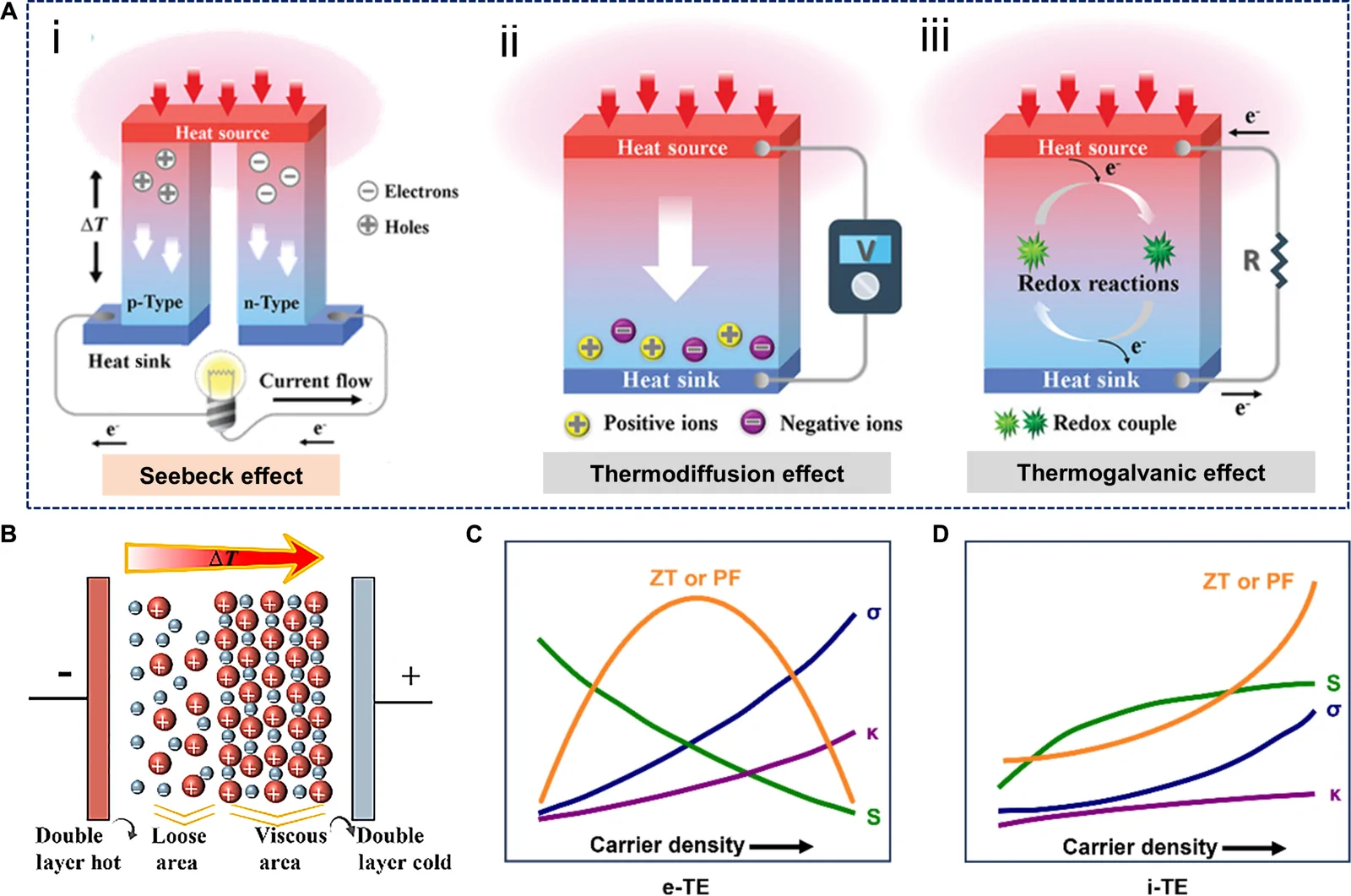
(A) Schematic diagram of (i) p-type and n-type e-TE materials, (ii) thermodiffusive i-TE materials, and (iii) thermogalvanic i-TE materials. Reproduced with permission [42]. Copyright 2023, John Wiley and Sons. (B) Internal structure of a thermodiffusive i-TE material in working mode. Reproduced with permission [65]. Copyright 2026, Royal Society of Chemistry. (C) Dependence of e-TE properties on carrier density. (D) Dependence of i-TE properties on carrier density. Reproduced with permission [7]. Copyright 2026, Elsevier.

#### Thermodiffusion effect

The i-TE effect primarily originates from the thermal diffusion of ions driven by a temperature gradient, i.e., the Soret effect [Fig. 2A(ii)] [57]. In cement-based materials, ions in the pore solution are the main charge carriers. When a ∆*T* exists across the 2 ends of the material, ions such as OH^−^, Na^+^, K^+^, and Ca^2+^ migrate under the ∆*T*. However, ions are normally featured with different migration speed (diffusion rate) due to distinctive size, activation energy, and Coulomb force [59]. Ions with different thermal diffusion coefficients migrate at unequal rates, leading to an asymmetric ion distribution along the ∆*T*. Driven by the ∆*T*, ions migrating to the cold end accumulate at the electrode/electrolyte interface and form an electric double layer (EDL) (Fig. 2B). According to the Gouy–Chapman–Stern theory, the electrical double layer consists of a compact layer and a diffuse layer [60], including a dense ion region near the cold side and a more dilute region with higher ion mobility near the hot side. This asymmetric distribution induces a gradient in electrochemical potential, producing a measurable thermal voltage [61]. Unlike e-TE materials, ions cannot cross the electrodes into the external circuit. Typically, the *S_i_* is defined as [40]:

$$S_{i}=-\frac{V\left(T_{H}\right)-V\left(T_{C}\right)}{T_{H}-T_{C}}$$ (1)

where *V*(*T_H_*) and *V*(*T*_C_) denote the voltages of the hot and cold electrodes, respectively. *T_H_* and *T*_C_ are the temperatures of the hot and cold electrodes, respectively. In i-TE systems, the sign of *S*_i_ depends on which type of ion dominates the thermal migration process. When the migration of anions (e.g., OH^−^) predominates, the cold end potential is lower and *S_i_* becomes negatively charged and exhibits n-type conduction, whereas when the migration of cations (e.g., Ca^2+^ and K^+^) predominates, the cold end potential is higher and *S_i_* becomes positively charged and exhibits p-type conduction.

#### Thermogalvanic effect

In addition to the thermodiffusion effect, thermally driven redox reactions can also generate TE potential [62] , as shown in Fig. 2A(iii). In an electrolyte system containing redox couples, when a ∆*T* exists across the 2 ends of the material, the ions participating in the redox reaction are oxidized at the anode and reduced at the cathode, causing the 2 electrodes to acquire different electrochemical potentials, thereby forming a continuous current in a closed circuit. A typical reaction can be expressed as:

$$O_{x}+{\mathrm{ne}}^{-}⇌R_{\mathrm{ed}}$$ (2)

where *O*_x_ and *R*_ed_ are the oxidized and reduced species, respectively, and *n* is the number of electrons transferred in the reaction.

The TE potential of the thermogalvanic effect originates from the entropy change of the redox reaction. Whether an oxidation or reduction reaction, the enthalpy (*H*) of the redox reaction can be expressed using the Gibbs free energy (*G*) as [63]:

$$\Delta G=\Delta H-T\Delta \hat{S}$$ (3)

where $\Delta \hat{S}$ is the entropy change of the redox reaction. By definition, the relationship between the electrode potential (*E*) and *G* is [64]:

ΔG=−nFE (4)

where *n* is the number of electrons transferred in the reaction and *F* is the Faraday constant.

Then, from the temperature derivative of the Nernst equation, the ionic Seebeck coefficient of thermogalvanic material (*S_tg_*) can be defined as [42]:

$$S_{\mathrm{tg}}=\frac{\mathrm{dE}}{\mathrm{dT}}=\frac{\Delta \hat{S}}{\mathrm{nF}}$$ (5)

This formula indicates that, unlike the thermodiffusion based on the Soret effect, the magnitude of the thermogalvanic potential is determined by the intrinsic entropy change of the redox couple, rather than the ion migration rate. Another notable characteristic of the thermogalvanic effect is its continuous output capability. In the thermodiffusion effect, once ions accumulate at the electrode interface to form a double layer, the i-TE potential tends to stabilize if no external circuit is connected. Once connected to an external circuit, the accumulated charge is rapidly discharged, causing the voltage to decay, thereby demonstrating the capacitor’s intermittent operating mode. In the thermogalvanic effect, redox reactions continue to take place at the hot and cold ends. As long as the temperature gradient is maintained, the electrode reactions continuously replenish the depleted charge carriers, thereby enabling a steady-state continuous current output [65].

### i-TE property evaluation parameters

#### Ionic conductivity (*σ_i_*)

*σ_i_* is a core parameter characterizing the ion migration capability in i-TECs, directly affecting the charge transport efficiency of TE conversion. It is defined as the ionic current density per unit electric field strength and can be expressed as [7]:

$$\sigma_{i}=\mu_{i}n_{i}q_{i}$$ (6)

where *μ*_i_, *n*_i_, and *q*_i_ represent the ion mobility, ion concentration, and ionic charge, respectively. In cement-based systems, the magnitude of *σ_i_* is jointly determined by the type of ionic charge carriers, the characteristics of the migration pathways, and the interaction between ions and the matrix. In addition, an increase in the electrolyte temperature may lead to the loss of stable cation–anion association, thereby enhancing the overall *σ_i_*. The temperature dependence of *σ_i_* can be expressed as follows [66]:

$$\sigma_{i}\left(T\right)=\sigma_{0}e^{\left(\frac{-E_{a}}{\mathrm{RT}}\right)}$$ (7)

where *σ_i_* (*T*) is the ionic conductivity at temperature *T*. *σ*_0_ is the conductivity at infinite high temperature. *E_ɑ_* is the activation energy for ion migration. *R* is the gas constant, with a value of approximately 8.314 J mol^−1^ K^−1^.

#### Thermal conductivity (*κ*)

*κ* is a key parameter determining the ability of i-TECs to maintain a temperature gradient. A low *κ* value can reduce the heat loss between the hot and cold ends, enhancing the thermodynamic driving force for TE conversion. Unlike crystalline ordered e-TE materials, the phonon propagation paths in i-TE materials are restricted, and diffuson thermal transport dominates (accounting for >80% of the total). The *κ* of i-TE materials is attributed to a dual-channel model, through the parallel integration of the phonon gas-like term and the diffuson term [67], expressed as:

$$\kappa =\kappa_{\mathrm{ph}}+\kappa_{\text{diff}}$$ (8)

$$\kappa_{\text{diff}}=\frac{n^{\frac{1}{3}}\kappa_{B}}{\pi }\int_{0}^{\omega }\frac{C\left(\omega \right)}{\kappa_{B}}\frac{g\left(\omega \right)}{3n}\mathrm{\omega d\omega}$$ (9)

where *κ_ph_* is the phonon thermal conductivity and *κ_diff_* is the diffuson thermal conductivity. *n* is the atomic number density. *g*(*ω*) is the density of diffuson vibrational states. *C*(*ω*) is the heat capacity. For cement-based materials, although their *κ_ph_* is typically high, the thermal conductivity can be effectively reduced by introducing porous structures or low-thermal-conductivity fillers.

#### TE figure of merit (*ZT*)

Although the TE performance of both e-TE and i-TE materials can be evaluated by *ZT*, their fundamental microscopic characteristics are different. The *ZT* is a dimensionless parameter, defined as follows [68]:

$$\mathrm{ZT}=\frac{\sigma \cdot S^{2}}{\kappa }T=\frac{\mathrm{PF}\cdot T}{\kappa }$$ (10)

where *S* is the TE potential or Seebeck coefficient. *σ* is the electrical conductivity. *PF* = *σ S*^2^ is the power factor. *T* is the absolute temperature. *κ* is the thermal conductivity. A high *ZT* value means that the material possesses low *κ* while maintaining good electrical properties, thus enabling a stable temperature gradient under a ∆*T* for efficient TE conversion. The *ZT_i_* value is a dimensionless indicator used to evaluate the energy conversion efficiency of TE materials. Its expression is as follows [7]:

$${\mathrm{ZT}}_{i}=\frac{\sigma_{i}\cdot S_{i}^{2}}{\kappa }T$$ (11)

where *S_i_* is the i-TE potential or ionic Seebeck coefficient. *σ_i_* is the ionic conductivity. TE parameters are closely related to the carrier density, as shown in Fig. 2C and D. In e-TE materials, *S* is negatively correlated with carrier concentration, while *σ* is positively correlated with it, and the total thermal conductivity (*κ_tot_*) is relatively independent of the carrier density. In contrast, in i-TE materials, there is no clear trade-off between the *S_i_* and *σ_i_*. In most cases, the *S_i_* of i-TE materials is essentially triggered by ion migration and the thermal reaction of redox couples. Therefore, a higher carrier concentration promotes an increase in accumulated charge, leading to a higher thermal voltage [69,70]. To achieve a higher *ZT_i_* value, the chemical composition can typically be tailored and the ion concentration and mobility optimized, thereby enhancing the *S_i_* [42,71].

Although Eq. (11) provides general guidance for the design of i-TE materials, recent advances in the i-TE field indicate that other parameters must be considered. Song et al. [72] argued that thermal power generation is primarily controlled by the Eastman entropy of ionic species, while ion diffusion determines the equilibration rate, resulting in a time-dependent voltage response that is closely coupled with the thermal field. Therefore, *S_i_* and *ZT_i_* in ionic systems should be interpreted within a dynamic rather than a steady-state framework to characterize the electrical conductivity. To better describe this coupling, they proposed a modified *ZT_i_* [72]:

$${\mathrm{ZT}}_{i}=\frac{{\mathrm{CS}}_{i}^{2}G_{T}}{\kappa \;\rho c}T$$ (12)

where *G_T_* is the interfacial thermal conductance, W m^−2^ K^−1^. *ρ* is the density, kg m^−3^. *C* is the quantity of accumulated net charge*. c* is the specific heat capacity, J kg^−1^ K^−1^. *T* is the absolute temperature, K. *σ_i_* is replaced by the product *C* × *G_T_*/(*ρc*), where *G_T_*/(*ρc*) represents the rate of electric energy generation over time. These newly introduced parameters have often been overlooked in previous studies, thus providing a new perspective for optimizing the performance of i-TECs.

Quantitative evaluation of energy conversion in i-TE materials also requires assessment of the thermodynamic efficiency (*η*). Based on thermodynamic cycle analysis, a 4-stage operation model [73]:

$$\eta =\frac{\Delta T}{T_{H}}\frac{{\mathrm{ZT}}_{i}}{2{\mathrm{ZT}}_{i}+\frac{10T}{T_{H}}-\frac{1}{2}{\mathrm{ZT}}_{i}\frac{\Delta T}{T_{H}}}$$ (13)

In comparison, the maximum efficiency for a traditional e-TE generator (TEG) is [74]:

$$\phi =\frac{\Delta T}{T_{H}}\frac{\sqrt{1+{\mathrm{ZT}}_{e}}-1}{\sqrt{1+{\mathrm{ZT}}_{e}}+\frac{T_{C}}{T_{H}}}$$ (14)

This implies that i-TE materials with a much lower *ZT_i_* due to poor *σ_i_* are difficult to achieve as a high satisfactory efficiency as classical TEGs [75,76].

## i-TE Properties of Cementitious Composite Materials

### Ordinary Portland cement

The TE properties of OPC are determined by the combined structural and chemical characteristics of their multiphase system (mineral phase, hydration products, and pore solution). Regarding composition, the primary minerals in OPC—tricalcium silicate (C_3_S, 50% to 60%), dicalcium silicate (C_2_S, 20% to 25%), tricalcium aluminate (C_3_A, 6% to 10%), and tetracalcium aluminoferrite (C_4_AF, 6% to 10%)—are all wide-bandgap insulators, possessing inherently poor e-TE properties [77]. Upon contact with water, these phases hydrate rapidly, forming a dense network composed primarily of calcium silicate hydrate (C-S-H) gel and calcium hydroxide (CH) (Fig. 3A). The C-S-H gel exhibits a disordered and nearly amorphous characteristic, similar to the 11-Å tobermorite [78] (Fig. 3B). Density functional theory (DFT) calculations confirm that CH [portlandite, Fig. 3C(i)] and C-S-H [Tobermorite-11, Fig. 3C(ii)] are wide-bandgap materials characterized by low lattice *κ* [79]. The bandgap of CH is 4.43 eV [Fig. 3C(iii)], while that of tobermorite-11 is 4.45 eV [Fig. 3C(iv)]. These values confirm that both are typical insulators, and their intrinsic carrier concentrations contribute negligibly to the macroscopic electrical conductivity. Further simulations reveal that as the water content within the tobermorite increases (from T9 Å to T14 Å), the bandgap narrows, and the *ZT* correspondingly increases from 0.983 to 1.272 [80] (Fig. 3D). Nevertheless, it is crucial to recognize that these are purely theoretical predictions at the crystal level.

**Fig. 3.**
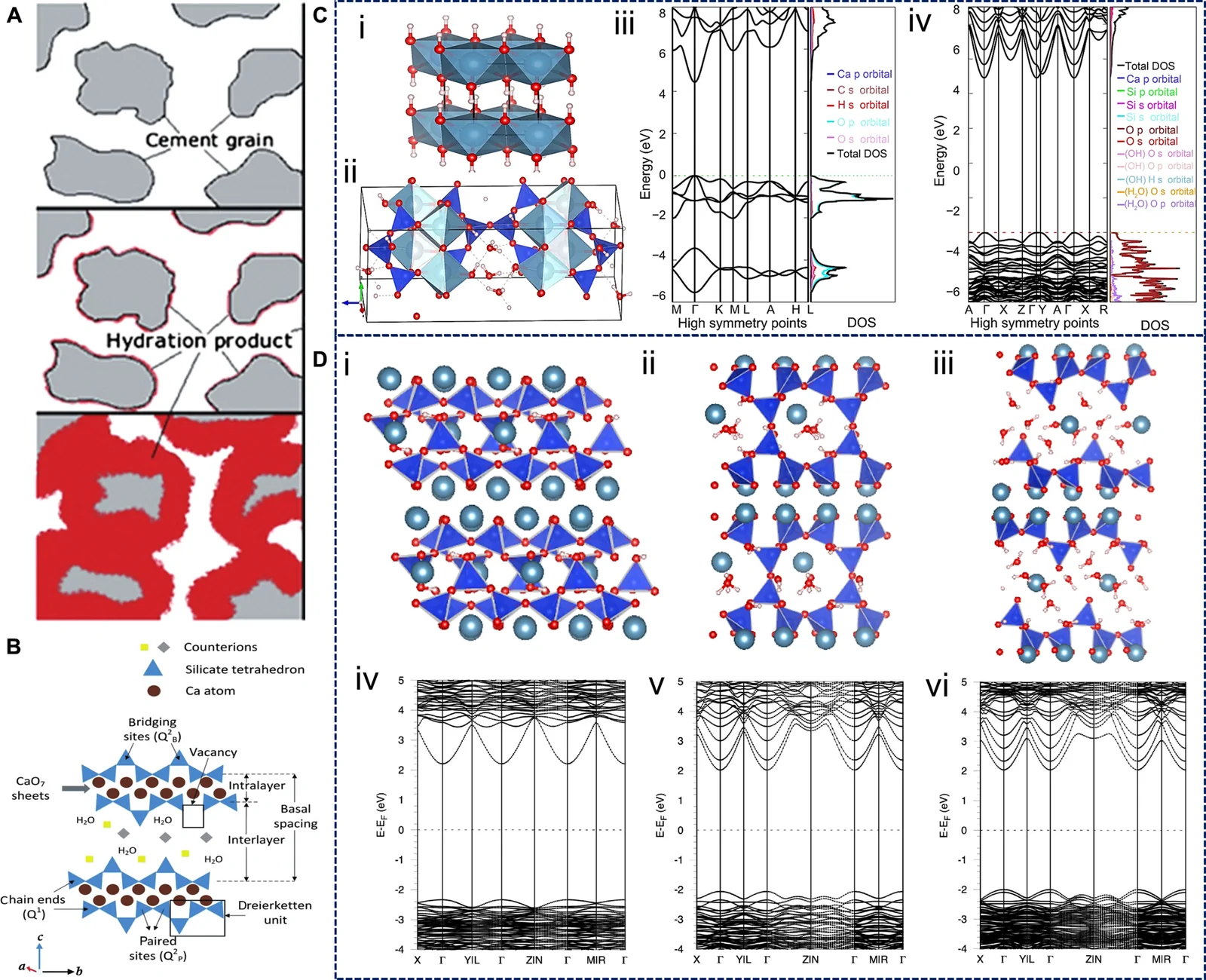
(A) Schematic diagram of the hydration process of OPC. Reproduced with permission [159]. Copyright 2015, Elsevier. (B) Schematic of the C-S-H structure from a defected tobermorite. Reproduced with permission [160]. Copyright 2013, American Chemical Society. (C) (i) Crystal structure of Ca(OH)_2_ (Ca, H, and O atoms are represented with gray, white, and red colors, respectively). (ii) Crystal structure of normal tobermorite-11 (Ca, H, Si, and O atoms are represented with gray, white, blue, and red colors, respectively). (iii and iv) DFT band structure and density of states of Ca(OH)_2_ and normal tobermorite-11 [79]. Copyright 2024, Elsevier. (D) Layered crystal structure of tobermorite mineral: (i) T9 Å [Ca_5_Si_6_O_16_(OH)_2_], (ii) T11 Å [Ca_4_Si_6_O_15_(OH)_2_‧5H_2_O], and (iii) T14 Å [Ca_5_Si_6_O_16_(OH)_2_‧7H_2_O]. The large gray spheres represent calcium atoms, the red spheres represent oxygen atoms, and the white spheres represent hydrogen atoms, respectively. The silicate tetrahedra are represented in blue, with the silicon atoms located in the center of the tetrahedra. Calculated electronic band structures of states per element in the unit cells of T9 Å (iv), T11 Å (v), and T14 Å (vi). The Fermi energy level (E_f_) is set at the zero mark and lies in the middle of the bandgap [80]. Copyright 2023, IOP Publishing.

In the actual hydrated OPC system, however, the presence of a porous structure and water shifts the dominant TE mechanism away from electronic transport and toward ionic transport and thermal diffusion. Following cement hydration, the pore solution is rich in OH^−^, K^+^, Na^+^, Ca^2+^, and other ions, with a pH typically maintained between 12.4 and 13.5 [46,81]. When a ∆*T* exists, these ions undergo selective thermal migration at different rates—a phenomenon known as the Soret effect. As the migration rate of OH^−^ is much higher than that of the metal cations in the system, OH^−^ preferentially accumulates at the cold end under a thermal gradient. This leads to spatial charge separation, where the cold side exhibits a lower electric potential and the hot side a higher one. This behavior endows OPC with a natural n-type i-TE characteristic. Consequently, the *S_i_* of cement paste is typically negative, with its magnitude ranging from −0.43 to −0.62 mV K^−1^ [48]. However, OPC suffers from inherently low *σ_i_* and relatively high *κ* (approximately 0.8 to 1.0 W m^−1^ K^−1^). Consequently, even though its *S_i_* far exceeds that of conventional e-TE materials, its overall *ZT* and *PF* remain extremely low, with *ZT* values on the order of 10^−7^ to 10^−5^. Therefore, the energy conversion efficiency of OPC is far too low for the direct harvesting of waste heat from buildings.

### Moisture

The influence of moisture (or humidity) on the i-TE properties of OPC is complex. Both the spatial distribution and the total amount of water in the pores directly govern the ion concentration, the connectivity of transport pathways, and even the dominant charge carrier type in thermal diffusion [82] (Fig. 4A).

**Fig. 4.**
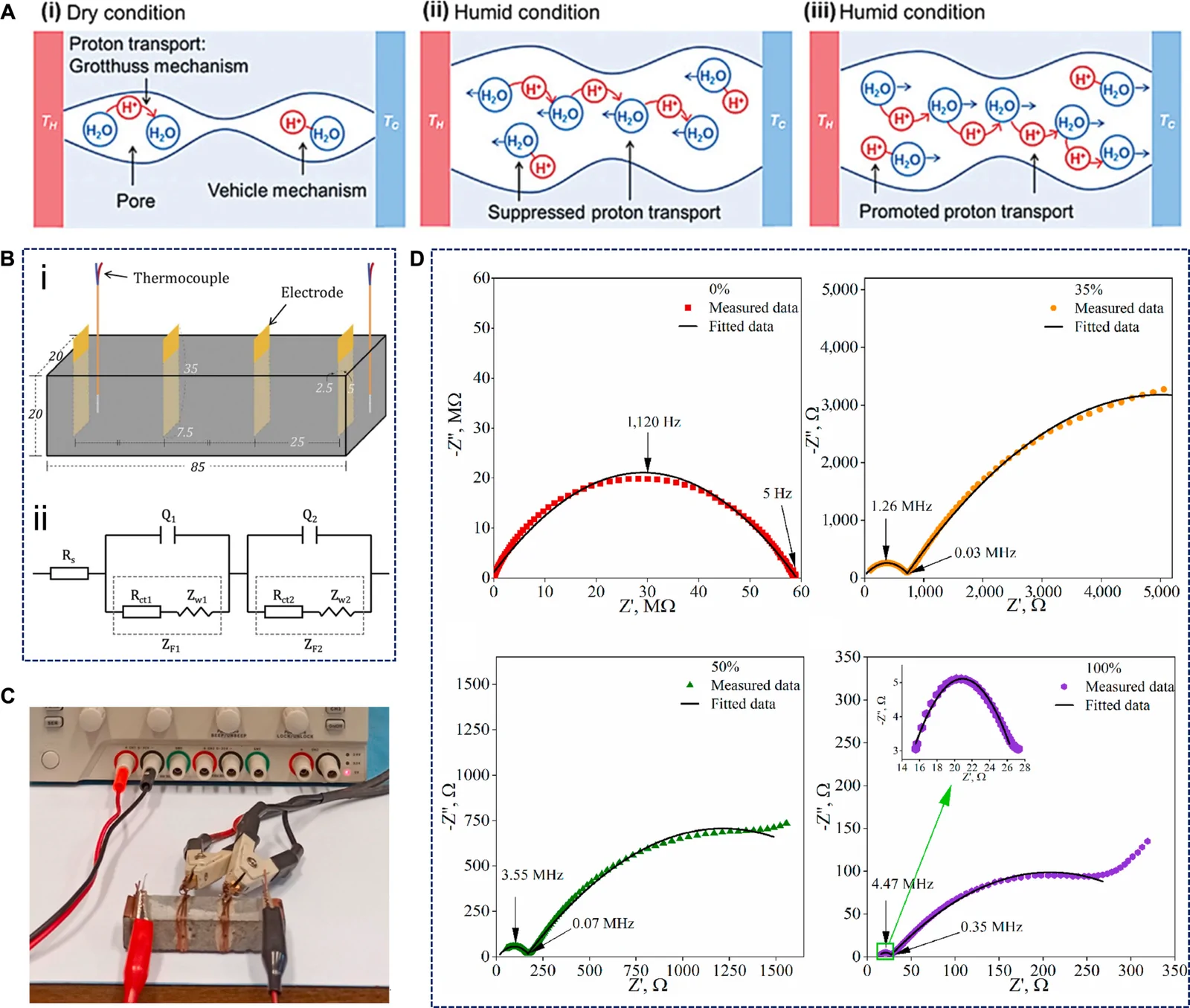
(A) Schematic illustration of ion and water transport under a temperature gradient through i-TE materials. (i) Dry condition. (ii) Semi-humid condition. (iii) Humid condition. Reproduced with permission [161]. Copyright 2018, Elsevier. (B) i-TEC sample for measurement of conductivity measurement sample: (i) schematic descriptions of sample and (ii) corresponding equivalent circuit for electrochemical system [85]. Copyright 2024, Elsevier. (C) Electrode layout for resistance measurements. Reproduced with permission [84]. Copyright 2024, Elsevier. (D) Nyquist plots for different saturation degrees. Reproduced with permission [84]. Copyright 2024, Elsevier.

The effect of moisture on ionic conduction is first manifested in the formation of conductive pathways. As illustrated in Fig. 4A(i), at low water content, the pore solution fails to form continuous channels across the capillary network. Consequently, ions remain trapped in isolated pores, and the *σ_i_* remains low. As the water content increases, interconnected pore-solution channels gradually develop, providing a medium that enables long-range migration of dissolved Ca^2+^, OH^−^, K^+^, and Na^+^, thereby increasing the *σ_i_* [Fig. 4A(ii)]. Experimental measurements indicate that the resistivity of dry cement matrices is approximately 10^8^ Ω cm, whereas in the water-saturated state it drops to around 10^3^ Ω cm [45]. This sharp decrease occurs because the loss of liquid water increases the resistivity of cement composites [83]. Electrochemical impedance spectroscopy (EIS) provides further microscopic evidence (Fig. 4B and C) [84,85]. With increasing moisture, the diameter of the high-frequency semicircle gradually decreases, whereas the low-frequency domain increases (Fig. 4D) [84]. Furthermore, increasing the water-to-cement ratio results in a higher volume of capillary pores after hardening, which indirectly raises the saturated water content and causes the *σ_i_* to increase exponentially [85]. This behavior stems from the dual-phase nature of OPC, where pores and a solid phase of limited conductivity form a complex network. In this network, when water is abundant, ionic conduction dominates the overall conductivity [86].

Similarly, the *S_i_* of OPC strongly depends on the water state, in agreement with the water-percolation theory [42]. Under water-saturated or high-humidity conditions, the OH^−^ concentration in the pore solution is high, and the thermal diffusion coefficient of OH^−^ is approximately 2 to 3 times that of the metal cations. Consequently, a temperature gradient drives OH^−^ preferentially from the hot end toward the cold end. The cold side thus becomes negatively charged owing to OH^−^ accumulation, yielding a typical n-type Seebeck effect, as illustrated in Fig. 4A(iii). Under these conditions, the *S_i_* of OPC can reach approximately −0.62 mV K^−1^ [48]. As the system gradually loses water, the pore solution becomes more concentrated, and some ions precipitate. The declining OH^−^ concentration weakens its dominant contribution to the overall thermopower, while the thermal diffusion contributions of cations such as Ca^2+^, K^+^, and Na^+^ become relatively more important. Eventually, the *S_i_* changes from negative to positive, and the OPC system exhibits p-type behavior [Fig. 4A(ii)]. When water is completely removed, ions lose their transport medium, and the thermal voltage arising from ionic thermal diffusion nearly vanishes. Any residual weak signal likely originates from very limited electronic conduction within the solid phases, causing the *S_i_* to drop to as low as 0.002 mV K^−1^ [48]. These observations collectively demonstrate that the i-TE response is fundamentally a cooperative outcome of the water-percolation network and competitive ion transport. On the one hand, the ionic conductors absorb the water and help dissociate the ions, which greatly enhance the ionic transport. On the other hand, the hydrovoltaic voltage caused by the water concentration gradient inside the materials also contributes to the overall thermopower measured [69,87].

Water content also regulates the *κ* of OPC. Although water itself conducts heat better than air, its contribution to the *κ_tot_* does not exhibit a simple positive correlation with water content when it is confined within nanopores and experiences strong interfacial scattering with the pore walls [88] . In general, as water saturation increases and air in the pores is displaced by water, phonon scattering at interfaces diminishes, and *κ* increases to some extent [89]. Valore [90] reported that the *κ* value increased about 6% with each 1% increment in the moisture of concrete. On the other hand, the highly porous structure formed by gel pores and capillary pores within the hydration products continuously scatters phonons, thereby maintaining the *κ* of both dry and saturated cement pastes at relatively low levels. Ambient humidity and temperature variations can also indirectly alter *κ* by influencing microcrack development, the formation of interfacial gaps, and porosity distribution, which further complicates the thermal transport behavior of OPC systems [91].

In summary, moisture profoundly influences the i-TE performance of OPC by simultaneously modulating their *σ_i_*, *S_i_*, and *κ*. At elevated humidity, water can enhance the i-TE properties by intensifying carrier scattering and polarization effects, as well as by creating a high density of interfacial defects [16,29]. However, excessive free water may degrade the mechanical properties and increase interfacial contact resistance, leading to performance deterioration over prolonged service [86]. Therefore, moisture control in OPC must strike a balance between efficient ion transport and the structural stability of the matrix.

### Pore structure

As cement hydration proceeds, a dense and tortuous pore network gradually develops within the cement matrix. The schematic diagram of ion transport in the pore structure is shown in Fig. 5A [92]. Parameters such as porosity, pore connectivity, and specific surface area (SSA) strongly influence both the transport efficiency of charge carriers and the selectivity of ion migration [49].

**Fig. 5.**
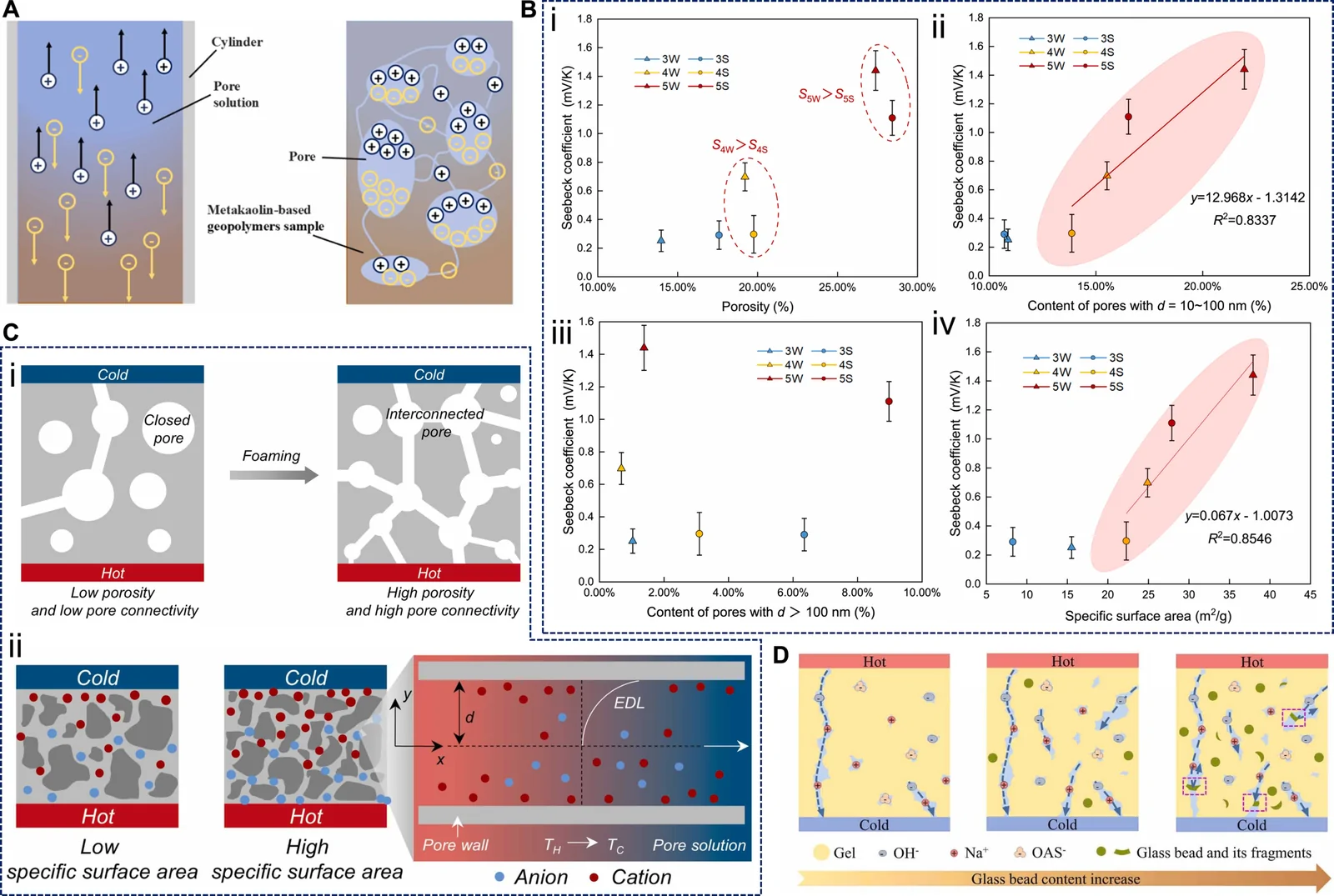
(A) Ion movement in pore solution and pores. Reproduced with permission [92]. Copyright 2025, Elsevier. (B) (i) The relationship of the *S_i_* with the porosity of the OPC. (ii) Relationship of the *S_i_* with the SSA of the OPC. The relationship of the *S_i_* with (iii) 10- to 100-nm pore content and (iv) >100-nm pore content. Reproduced with permission [49]. Copyright 2024, Elsevier. (C) (i) Schematic diagram of morphological benefits of the unique architecture of the samples. (ii) Schematic diagram of the desorption process of ions in the cement matrix. Reproduced with permission [93]. Copyright 2025, Elsevier. (D) Schematic diagram of the TE mechanism of MKG with different glass bead contents. Reproduced with permission [100]. Copyright 2025, Elsevier.

Porosity determines the abundance of the ionic transport medium. Cui and Wei [49] adjusted the pore structure in OPC by varying the water-to-cement ratio. They found a strong positive correlation between porosity and the *σ_i_* of hardened cement paste [Fig. 5B(i)]. When the porosity increases from the range of 13% to 17% to 27% to 28%, the *σ_i_* can rise by more than 145%, thereby providing the necessary channel capacity for large-scale thermal diffusion of ions under a temperature gradient. Excessively low porosity (<15%) severely disrupts the continuity of ion migration pathways. Consequently, even when the ion concentration in the pore solution is high, the *S_i_* remains below 0.5 mV K^−1^ [49]. It is important to note that the beneficial effect of high porosity described above applies specifically to neat cement systems without ionic conductive fillers. With the addition of ionic conductive fillers, favorable ion transport pathways can be formed even at relatively low porosity. In this case, the i-TE performance may become less dependent on matrix porosity, with water-filled ionic channels playing the dominant role.

Concurrently, improved pore connectivity optimizes channel continuity, reducing energy dissipation caused by pathway discontinuities or isolated pores during ionic transport [93]. Higher pore connectivity, often characterized by a larger critical pore diameter, promotes smoother migration of metal cations and OH^−^. Therefore, a rational pore size distribution is essential for achieving i-TE selectivity. Studies indicated that fine pores in the 10- to 100-nm range are optimal for enhancing the i-TE effect, and a higher proportion of such pores leads to a larger *S_i_* [49] [Fig. 5B(ii)]. Mesopores (2 to 50 nm), which are formed by gel pores and capillary pores, offer multiple synergistic advantages in both ion transport and thermal management. On the one hand, a dense mesoporous network effectively enhances phonon scattering at interfaces, thereby greatly lowering *κ* without substantially compromising *σ_i_* [94]. On the other hand, nanoscale channels create a confinement effect. The stronger constraints imposed by pore walls on ion migration improve the directionality of ion transport, thereby increasing ion or carrier mobility [95]. For instance, the addition of the surfactant TX10 can concentrate the pore size distribution around 28.3 nm, which promotes uniform dispersion of functional fillers and optimizes the interaction between ions and pore walls [96]. In contrast, when coarse pores (>100 nm) dominate, the self-desiccation effect leads to discontinuous contraction of the pore solution within the channels, which makes ion migration more disordered and causes the *S_i_* to drop [Fig. 5B(iii)] [97].

Furthermore, the SSA provides a driving force for enhancing the *S_i_*, as it strengthens the EDL at the solid–liquid interface. A positive correlation exists between SSA and the *S_i_* (*R*^2^ = 0.85), as shown in Fig. 5B(iv). When the SSA increases from the range of 15 to 25 m^2^ g^−1^ to 22 to 38 m^2^ g^−1^, the *S_i_* rises markedly. In particular, when the SSA exceeds 30 m^2^ g^−1^, the coordinative adsorption of high-valence cations such as Ca^2+^ onto the pore walls is markedly enhanced, which restricts their thermal migration. Meanwhile, OH^−^ can still move rapidly through the fine pore channels. This maximizes the difference in migration rates between cations and anions, allowing the *S_i_* to reach 1.44 mV K^−1^. In contrast, when the SSA is below 20 m^2^ g^−1^, the EDL effect is weak, the difference in ion mobilities is small, and the *S_i_* typically falls below 0.8 mV K^−1^ [49].

This finding clearly demonstrates that higher porosity alone does not guarantee efficient TE conversion. Instead, the key lies in constructing ion transport pathways that exhibit low tortuosity and high connectivity [Fig. 5C(i)]. Meanwhile, the increased SSA considerably enlarges the contact interface between pore walls and pore solution, allowing surface charges to promote the generation of higher density EDL structures [98] [Fig. 5C(ii)]. According to EDL theory in micro/nanochannels, a reduction in channel radius elevates the EDL potential, which contributes to an increase in the *S_i_* [99]. However, an increase in the pore structure will lead to an increase in defects, which not only hinders the ion migration and disrupts the matrix continuity but also enhances the scattering of charge carriers at the interface, thereby reducing the efficiency of ion diffusion (Fig. 5D) [100]. Therefore, optimizing the fine-pore fraction and SSA is effectively equivalent to constructing countless selective ion-transport units within the OPC, which amplifies the asymmetry of thermal diffusion between cations and anions at the microscopic scale.

Beyond influencing ion transport, pore structure also plays a critical role in regulating *κ*. In general, as porosity increases or pores become larger, the *κ* of cement-based materials tends to decrease owing to enhanced phonon scattering and the low *κ* of air in the pores [101]. The reduction in *κ* indirectly enhances the TE output, thereby stabilizing the local thermal gradient, which is crucial for the continuous energy conversion process [102]. Moreover, while a highly connected pore network provides fast channels for ionic conduction, it may also create low-resistance pathways for heat flow across the matrix, partially offsetting the thermal insulation benefits gained from higher porosity. As discussed in the previous section, moisture content influences matrix densification and porosity distribution. Furthermore, the presence of water in nanopores can introduce complex interfacial thermal resistance effects, which further affect *κ* at both microscopic and macroscopic scales [91,103].

In summary, the regulation of the i-TE effect by pore structure is governed jointly by porosity, pore connectivity, interfacial confinement, and electrochemical selectivity. A well-designed pore structure offers the prospect of maximizing thermopower output while maintaining both high *σ_i_* and low *κ*. However, the i-TE responses of the cement system are dependent on the presence of water. The pore solution must maintain an adequate saturation level to sustain ion transport, ion mobility, and the formation of the EDL. This moisture dependence represents a major challenge in practical applications, as rapid drying markedly reduces *σ_i_*. Therefore, water-retaining strategies such as hydrophobic coatings, self-healing encapsulation, or adding hygroscopic additives are crucial for long-term performance.

### Cementitious composite materials

In recent years, engineered cementitious composites (ECCs) have attracted considerable research interest as multifunctional smart building materials, owing to their excellent mechanical properties, low density, low *κ*, and self-healing capability [104–106]. In particular, when mineral admixtures such as fly ash and silica fume are incorporated into ECC to create a highly porous ultra-lightweight ECC (ULW-ECC), the resulting material exhibits a markedly stronger i-TE effect than OPC [98,107]. Ran et al. [108] prepared an ULW-ECC using fly ash cenospheres [FACs; Fig. 6A(i)] and calcined petroleum coke [CPC; Fig. 6A(ii)], achieving an *S_i_* of up to 4.06 mV K^−1^, which is far higher than that of OPC. This enhancement arises because the pozzolanic reaction of the mineral admixtures actively reduces the OH^−^ concentration in the pore solution, thereby allowing metal cations such as Ca^2+^, Na^+^, and K^+^ to become the dominant charge carriers and generating a p-type i-TE effect. Furthermore, by incorporating an air-entraining admixture (AEA) into ECC, they further raised the *S_i_* to 4.06 mV K^−1^ [98] and observed a positive correlation between the *S_i_* and porosity. As shown in Fig. 6B(i), the porosity of ULW-ECC with AEA exceeds 60%, which is substantially higher than the 22.79% of OPC. This improvement results from the porous conductive structure created by AEA within the ULW-ECC. This structure not only markedly lowers the *κ* but also increases the SSA of the pore solution, thereby providing more pathways for ionic thermal diffusion [98]. Additionally, leaching treatment further enhances the *S_i_*, as illustrated in Fig. 6B(ii) and (iii). This is because leaching removes more OH^−^, thereby reinforcing the dominant role of cations and increasing the TE voltage, whereas drying diminishes the ionic contribution [107] (Fig. 6C). Overall, ECC exhibits great promise as an efficient, multifunctional i-TE material, owing to its low *κ*, high porosity, and unique ability to regulate the ionic species in the pore solution.

**Fig. 6.**
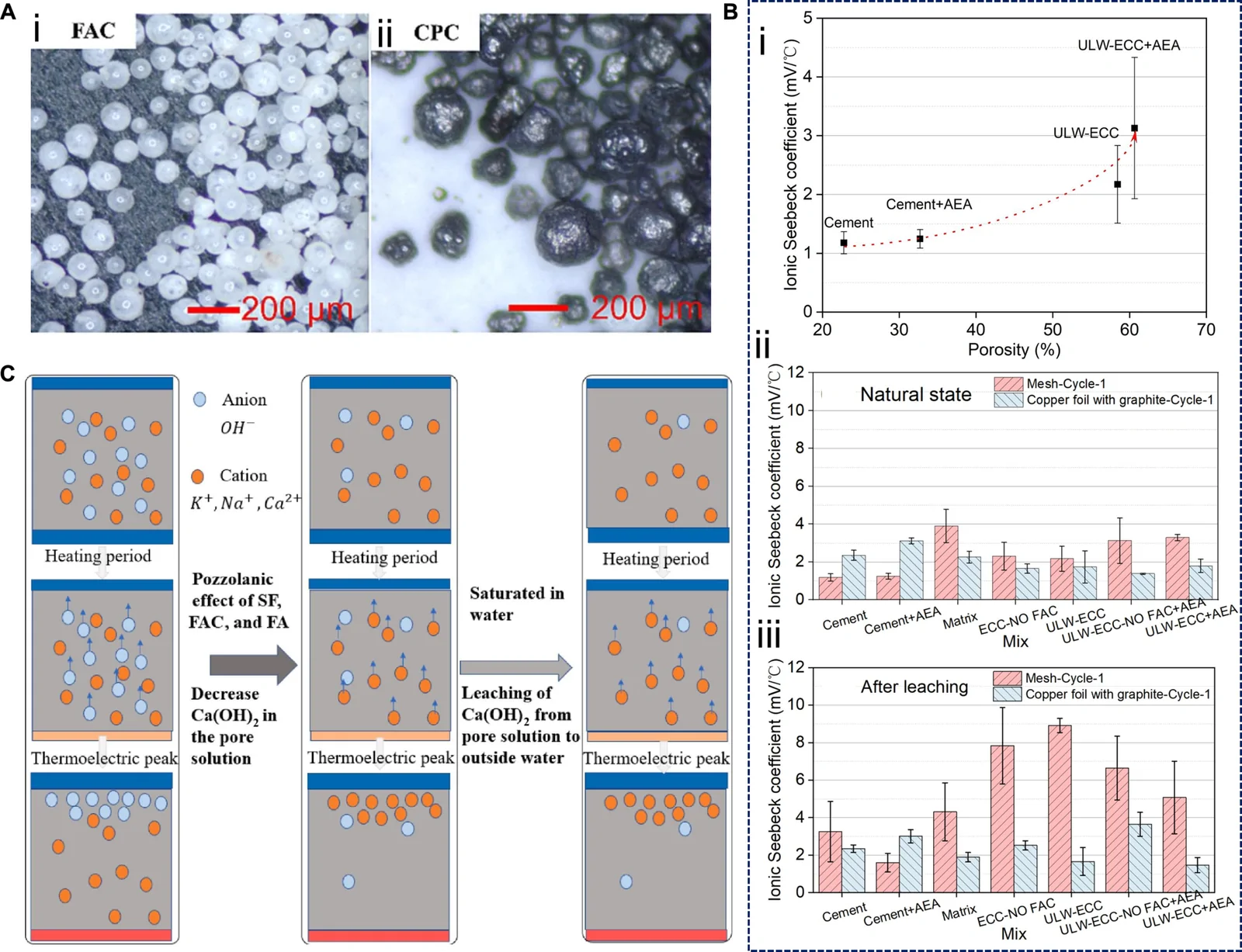
(A) Morphologies observed by digital microscopy of (i) FAC and (ii) CPC. Reproduced with permission [108]. Copyright 2023, Elsevier. (B) (i) Effect of porosity on the *S_i_*. The *S_i_* of the ULW-ECC composite: (ii) natural state (iii) after leaching [98]. Copyright 2024, Elsevier. (C) Cooperative working mechanism of ULW-ECCs [107]. Copyright 2024, Elsevier.

Furthermore, ultra-high performance concrete (UHPC) provides an ideal platform for integrating energy harvesting into load-bearing structures due to its superior mechanical properties and durability. Current studies indicated that by incorporating conductive fillers, the *σ* and *S* of UHPC can be effectively enhanced, with the optimal *ZT* value reaching the order of 10^−7^ [109–113]. However, its inherently dense microstructure and discontinuous pore network are unfavorable for ionic transport. Unlike the OPC system, UHPC contains steel fibers, which enhance mechanical properties through crack bridging. It has been shown that the continuous conductive network formed by fibers not only provides pathways for electron transport but also facilitates the migration of ions within the matrix [104]. It indicates that the fiber–matrix interface can serve as a path for ion transport, but it is still unclear how the steel fibers affect the ion transport within the UHPC. Meanwhile, steel fibers can bridge microcracks, which help maintain the continuity of pore solution channels [114,115]. On the other hand, the fiber–matrix interface itself can serve as a preferential pathway for water transport, potentially accelerating drying if fibers are poorly bonded. This viewpoint still requires experimental evidence to clarify this issue. Yoo et al. [114] believe that the amount, length, and shape of the fibers have a crucial influence on the crack development in UHPC. Therefore, steel fibers will become an important design parameter affecting the continuity of ion channels in UHPC.

Moreover, UHPC also has the potential to overcome its transport limitations through post-treatment methods (such as water gel vacuum impregnation, introduction of ordered ion channels, or addition of air-entraining agents), thereby reconciling its dense structure with the pore structure requirements described in the previous section. These measures are expected to enhance the i-TE effect of UHPC, thereby achieving the integration of structure and function.

### Long-term stability and service life considerations

While the immediate i-TE performance of cement-based composites has been extensively characterized, the stability and repeatability of this performance during the service life of building components remain critical concerns for practical deployment [116]. Under actual conditions, environmental factors such as dry–wet cycles, carbonation, and freeze–thaw cycles can have an impact on the moisture and ion transport of cement-based materials. Wetting–drying cycles regulate ion transport by altering the internal relative humidity and pore water saturation of concrete, coupling convective and diffusive transport [117]. During the drying stage, internal moisture migrates outward, generating negative pore pressure and capillary suction. When re-wetted, external ionic solutions are rapidly drawn into the surface layer, resulting in convection-dominated ion transport. In addition, cyclic wetting–drying deteriorates the micropore structure and interfacial transition zone (ITZ), enhances pore connectivity, and thereby further facilitates ion migration. For carbonation, cement-based materials undergo reactions between atmospheric CO_2_ and cement hydration products, which lower the pH of the pore solution and consume OH^−^ ions [118]. This change may shift *S_i_* toward p-type behavior, since the contribution of cations becomes relatively more dominant. Similarly, when cement-based materials are exposed to rainwater or groundwater for a long time, it may lead to the loss of alkali metal ions and OH^−^. Meanwhile, the permeation of chloride ions or sulfates in the environment may introduce competitive ions, thereby altering the kinetic behavior of the Soret effect. Repeated freeze–thaw cycles can lead to microcrack propagation and coarsening of the pore structure [119]. Although microcracks may initially enhance ion transport pathways, they also accelerate moisture loss and carbon dioxide ingress, thereby degrading performance. Moreover, during the low-temperature freezing stage, ice crystals formed in the pores occupy the liquid-phase space and temporarily hinder ion migration [120]. Therefore, long-term cyclic testing and verification in actual service environments are urgent issues to be addressed in this field.

## i-TE Properties of Other Cementitious Materials

The pore solution of the OPC system is typically composed of ions such as OH^−^, Ca^2+^, K^+^, and Na^+^. This chemical composition determines the upper limit of the adjustable carrier types and concentration ranges. More importantly, the i-TE performance of OPC is highly sensitive to humidity. Once the material dries out, the i-TE response mechanism tends to fail. It has been reported that the *σ_i_* of OPC can decrease by 15% to 20% within a 90-d curing [84]. Therefore, researchers have attempted to break out of the inherent framework of OPC and by selecting alternative cement types or designing new cementitious systems with different ion types, water products, and pore solutions, with the aim of enhancing the i-TE properties. The statistical results are shown in Table 1.

**Table 1.** i-TE performance of cement-based composite materials

Matrix	Functional filler/modification	Treatment method	*σ_i_* (S m^−1^)	*S_i_* (mV K^−1^)	*κ* (W m^−1^ K^−1^)	*PF* (μWm^−1^ K^−2^)	*ZT*	Test conditions	28d Compressive strength (MPa)	Year	Ref.
Cement		-	-	−0.62	-	-	-	Saturated state, Δ*T* = 30–50 K	-	2023	[48]
		Ion leaching 72 h	0.053	1.06	-	0.060	-		-	2023	[48]
	10 wt % Ca_12_Al_14_O_33_/CB	Heat treatment at 1,073 K	480	0.025 (380 K)	1.57	0.24 (950 K)	0.16 × 10^−3^ (950 K)	Dry state	-	2020	[164]
	0.15 wt % rGO	Ion leaching 72 h	1.9 × 10^−2^	1.859	-	6.55 × 10^−2^	-	Saturated state	-	2022	[47]
		Ion leaching 168 h	0.103	1.44	-	0.178	-	Saturated state	-	2024	[49]
	PVA-Ca(OH)_2_ hydrogel	Ice-template method	1.07 × 10^−1^	−40.5	0.77	168.6	6.6 × 10^−2^	Hydrogel hydrated state, Δ*T* = 10 K	15.1	2025	[54]
		Lemon juice impregnating for 48 h	-	1.22	-	-	-	As-received	38.14	2025	[133]
	0.25 wt % SDS	Ion leaching 72 h	0.213	1.57	0.93	0.525	1.9 × 10^−4^		4.2	2025	[93]
	PVA hydrogel/KOH		-	7.35	-	-	-	Saturated state	30.36	2025	[136]
	2.0 wt % PVA + 0.5 wt % SA	Ca (NO_3_)_2_ immersion	0.0732	2.96	-	0.62	-	Saturated state, Δ*T* = 45 K	-	2026	[134]
	2 wt % PVA + K_3_[Fe (CN)_6_]		1.6	11.25	0.69	17.1	7.8 × 10^−3^	Hydrated state, Δ*T* = 10 K	49.9	2026	[146]
	0.1 wt % Biomass-derived cellulose fibers		2.58 × 10^−3^	9.39	-	0.228	2.4 × 10^−4^	Saturated state	27.3	2026	[165]
Ultra-lightweight engineered cementitious composites (ULW-ECC)	10 wt % calcined petroleum coke (CPC)		8.24 × 10^−4^	4.060	0.943	1.36 × 10^−2^	4.51 × 10^−6^	As-received	76.32	2023	[108]
	2 wt % AEA	Leaching		≈5	0.28			As-received	64.5	2024	[98]
	1.0 vol % CF	Saturation treatment	4.83 × 10^−4^	5.932	0.478	1.7 × 10^−2^	9.17 × 10^−6^	Saturated state	31.9	2024	[107]
Calcium sulfoaluminate (CSA) cement			1 × 10^−5^	5.3	-	-	-	As-received	-	2024	[121]
Magnesium phosphate cement (MPC)	5.0 wt % CB		104.17	11.16	-	1513	0.523	As-received, Δ*T* = 20 K	-	2025	[123]
	5 wt % SiC		0.025–0.03	+7.10	0.5–0.6	1.2–1.4	0.0002–0.0003	As-received	45.5	2026	[124]
	10 wt % Acrylamide (AM)		0.035–0.04	−3.85	0.8–0.9	0.5–0.6	0.0007–0.0008	In situ polymerization	36.0		
Alkali-activated cement (AAC)	2 wt % MWCNTs		-	0.0226	-	4.8 × 10^–3^	-	As-received	-	2021	[166]
	1.0 wt % SWCNT		1,660	0.0158	-	0.414	-	As-received	45	2021	[167]
	Alkali-activated slag + Fe-rich copper slag sand		-	3.013	-	4.5 × 10^–6^	1.594 × 10^−3^	As-received	36	2026	[168]
Geopolymer		70 °C curing	-	−0.0151	-	-	-	As-received	-	2020	[131]
	10% SiC		-	−0.0293	-	-	-	As-received	-	2021	[169]
			1.41 × 10^−3^	2.17	0.2	-	5.3 × 10^−4^	As-received	-	2021	[130]
	5 wt % MnO_2_		-	−4.27	-	-	-	As-received	36.8	2023	[170]
	0.65 wt % P-type CNTF		1.64 × 10^5^	0.0452		335.8		As-received, Δ*T* = 50 K		2024	[143]
	0.65 wt % N-type CNTF		1.16 × 10^5^	−0.0384		171.4					
	30 wt % Red Mud (RM) + 2 wt % nano-ZnO		401.75 × 10^−3^	0.02906	0.734	-	54.4 × 10^−7^	As-received	63.9	2024	[171]
	50% Tinoca Mine residue		1.45 × 10^−3^	−2.76±0.069	0.32	-	9.56 × 10^−7^	As-received	10.9	2024	[172]
	75 wt % copper slag aggregate		3.0 × 10^−4^	0.570	0.58	-	1.7 × 10^−7^	As-received	-	2024	[132]
	90 wt % FA + 10 wt % GGBFS		-	1.353	-	-	-	As-received	36.1	2025	[139]
	0.5 wt % MWCNT + 6 wt % RM		-	2.544	-	-	5.1 × 10^−7^	As-received	63.13	2025	[173]
		Soaked in deionized water	0.58 × 10^−3^	1.700	0.71	1.68 × 10^−9^	0.59 × 10^−7^	Saturated state	-	2025	[92]
	5 wt % Fe_2_O_3_ + GNPs		-	1.092	1.2	0.84	2.0 × 10^−4^	As-received	33.3	2025	[174]
	5 wt % MnO_2_ + PVA fibers		0.004	−5.470	0.79	-	2.74 × 10^−5^	As-received	65.6	2025	[175]
	Glass bead			1.24		0.49 × 10^−6^	1.50 × 10^−5^ (298 K)	As-received		2025	[100]
		Pt electrode		2.01				As-received		2026	[128]
	5 wt % graphite powder+ 5 wt % flake graphite + 0.8%carbon fiber		0.35	0.0417	2.39	6.09 × 10^−4^	7.63 × 10^−8^	As-received	33.8	2026	[176]
	0.01 wt % CF		6.87 × 10^−4^	3.46	0.7276	-	2.82 × 10^−7^	As-received	>40	2026	[140]

### Other types of cement

Sarabia-Riquelme et al. [121] first employed calcium sulfoaluminate (CSA) cement as a replacement for OPC. The hydration products of CSA are mainly ettringite and monosulfate. In addition to the common ions OH^−^, K^+^, and Na^+^, the pore solution also contains a high concentration of Al(OH)_4_^−^, which is introduced by the hydration of sulfur-bearing mineral phases. This additional ionic species provides alternative charge carriers for i-TE that are distinct from OH^−^. The results show that fully hydrated CSA exhibits an *S*_i_ of approximately 5.3 mV K^−1^, and its thermal stability is about 20% higher than that of OPC. This means that under the same ∆*T* and age, the i-TE voltage of CSA decays more slowly. However, the ionic size of Al(OH)_4_^−^ is considerably larger than that of OH^−^, and its mobility in aqueous solution is relatively lower. Consequently, the i-TE response of the CSA system is noticeably slower than that of OPC, exhibiting a longer voltage relaxation time.

Katoite (Ca_3_Al_2_(OH)_12_), a stable hydration product of calcium aluminate cement (CAC), offers a unique structural perspective for i-TECs, as shown in Fig. 7A(i). The katoite structure contains abundant OH^−^ groups, but its crystal framework places these groups in a chemical environment that is distinctly different from that in the C-S-H gel of OPC [122]. First-principles calculations reveal that aliovalent substitution of Mg^2+^ for Al^3+^ can substantially modulate both the electronic band structure and the ionic defect chemistry of katoite. Specifically, hole doping raises the *ZT* to 0.6, whereas electron doping yields a *ZT* of 0.31 [Fig. 7A(ii) and (iii)]. This enhancement is attributed to the higher thermal conductivity, higher thermal diffusivity, and greater thermal energy storage capacity of katoite compared with cement.

**Fig. 7.**
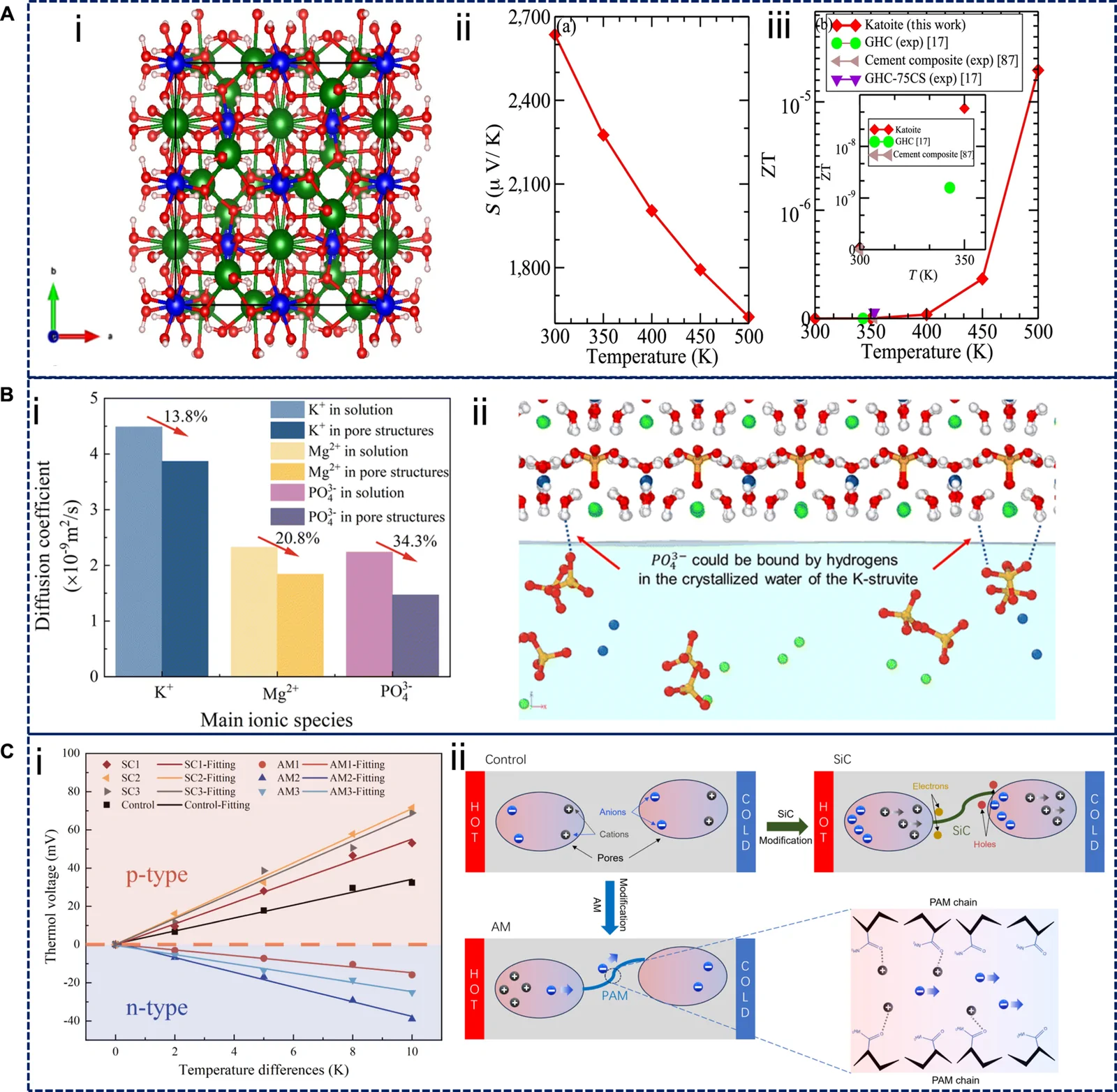
(A) (i) Crystal structure of Ca_3_Al_2_(OH)_12_ (Al, Ca, O, and H are represented with blue, green, red, and white colors, respectively). Intrinsic TE properties of katoite Ca_3_Al_2_(OH)_12_: (ii) *S* and (iii) TE figure of merit [122]. Copyright 2025, Elsevier. (B) (i) Calculated mean square displacement (MSD) results of the dominant ions within and outside of the MPC pore structure. (ii) Schematic illustration of the PO_4_^3−^ bound by hydrogen atoms in the K-struvite. Reproduced with permission [123]. Copyright 2025, Royal Society of Chemistry. (C) Results of MPC thermopotential tests: (i) Fitted curves of the generated TE voltage difference corresponding to the associated temperature difference. (ii) Conceptual diagram of the mechanism of the TE effect for different modifications of the MPCs. Reproduced with permission [124]. Copyright 2026, Elsevier.

Magnesium phosphate cement (MPC) can simultaneously introduce both metal cations and anions, thereby improving the stability of its hydration products. Studies indicated that the main hydration product of MPC is K-struvite. Its pore solution is rich in K^+^ (79.8 mM), Mg^2+^ (5.6 mM), and PO_4_^3−^ (74.8 mM), whereas the OH^−^ concentration is markedly lower than that in OPC systems [123]. As a high-valence oxyanion, PO_4_^3−^ forms hydrogen bonds with water molecules in the K-struvite structure. This imposes additional constraints on anion migration and thereby amplifies the mobility difference between anions and cations under a temperature gradient (Fig. 7B). Building on this, silicon carbide (SiC) and in situ polymerization of acrylamide (AM) were separately used to modify MPC, successfully yielding high-performance i-TECs with both p-type (+7.10 mV K^−1^) and n-type (−3.85 mV K^−1^) characteristics [124], as shown in Fig. 7C. This is because the SiC surface can act as a carrier trapping and transport site, whereas polyacrylamide (PAM) preferentially adsorb cations, together creating exclusive transport pathways for anions [Fig. 7C(ii)].

The studies described above demonstrate that by replacing the cementitious system, one can fundamentally alter the chemical composition of the pore solution and the dominant ionic species, thereby reducing the reliance on OH^−^ as the sole charge carrier and broadening the scope of the i-TE response.

### Geopolymers

Geopolymers have recently attracted considerable interest for i-TE energy harvesting in buildings, owing to their low carbon emissions, wide raw material availability, and favorable ionic conduction properties [125,126]. The formation of geopolymers proceeds through a dissolution–polycondensation process of aluminosilicate sources (e.g., fly ash, metakaolin, or slag) under the action of alkaline activators, ultimately yielding a 3-dimensional (3D) network of sodium aluminosilicate hydrate (N-A-S-H) gel. This gel is built from [SiO_4_]^−^ and [AlO_4_]^−^ tetrahedra linked by oxygen atoms [127] (Fig. 8A). X-ray photoelectron spectroscopy (XPS) results indicate that the Fermi level of the geopolymer lies within its conduction band, confirming its distinctly n-type character [128] (Fig. 8B). Although the n-type response of saturated N-A-S-H gel appears superficially similar to that of the C-S-H gel in hydrated OPC, a fundamental difference exists between the two. As illustrated in Fig. 8C(i), the C-S-H gel possesses a layered structure in which a CaO backbone is flanked by silicate chains. Its Q^3^ silicate tetrahedral units contain numerous intrinsic defects, including nanopores, microcracks, and weak interfaces between different hydration phases [129]. In contrast, the N-A-S-H gel is a single-phase continuous material, exhibiting fewer macroscopic interfaces between phases. This structural homogeneity greatly reduces interfacial scattering of charge carriers during transport, thereby enhancing ionic diffusion efficiency [130]. Moreover, the alkaline activation process employs concentrated NaOH, KOH, or water glass as activators. Consequently, the pore solution of geopolymers is mainly composed of Na^+^, K^+^, OH^−^, and SiO_3_^2−^, with the concentrations of Na^+^ and K^+^ far exceeding those in OPC. It has been reported that the total ion concentration in the pore solution of fly ash–slag geopolymers can reach 320 to 3,790 mg l^−1^, compared with only about 216 mg l^−1^ for OPC [130] [Fig. 8C(ii)].

**Fig. 8.**
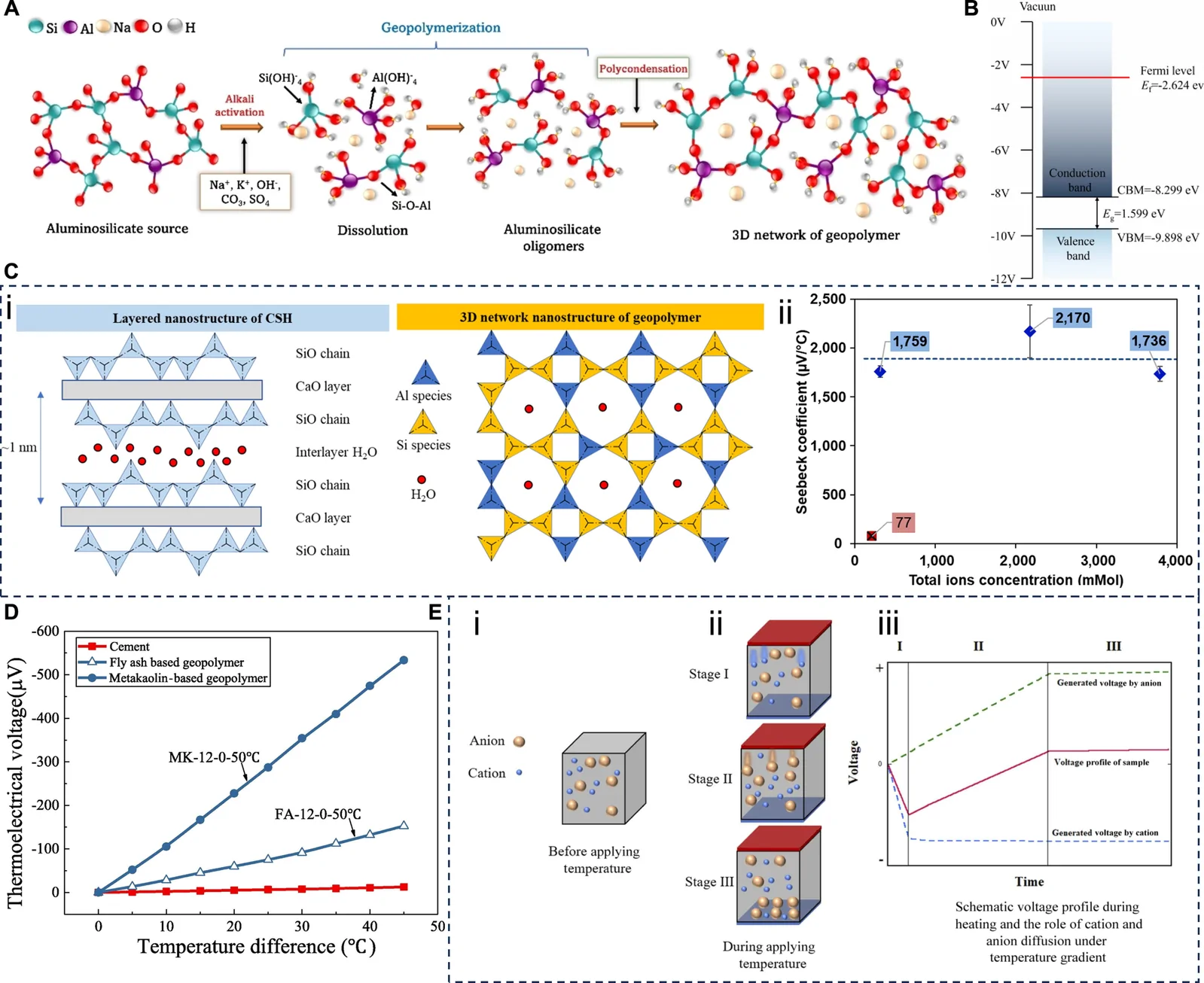
(A) Schematic representation of the process of geopolymerization associated with geopolymer synthesis. Reproduced with permission [162]. Copyright 2025, John Wiley and Sons. (B) Electronic band structure of the geopolymer. Reproduced with permission [128]. Copyright 2026, Elsevier. (C) (i) Comparison of nanostructure of C-S-H and geopolymer. (ii) Relation of *S_i_* and total ion concentration of metakaolin-based geopolymers and OPC. Reproduced with permission [130]. Copyright 2021, Elsevier. (D) Seebeck voltage versus temperature difference for geopolymers and cement. Reproduced with permission [131]. Copyright 2020, Elsevier. (E) Schematic view of (i) ions in the initial stage before applying Δ*T*, (ii) diffusion of ions during different stages of voltage profile, and (iii) the role of different ion diffusion on voltage profile. Reproduced with permission [132]. Copyright 2024, Elsevier.

Benefiting from the optimized ion concentration and reduced interfacial scattering associated with the hydration products, fly-ash- and metakaolin-based geopolymers can achieve an *S_i_* of −0.0151 mV K^−1^ [131], far exceeding that of OPC (Fig. 8D). Barzegar et al. [132] further analyzed the schematic diffusion of cations and anions at different stages of applying a temperature gradient (Fig. 8E). Stage I corresponds to the generation of a negative voltage as alkali metal cations migrate away from the hot electrode. Stage II involves the release of OH^−^ from the hot electrode, which reduces the potential difference between the electrodes. Stage III is the accumulation of OH^−^ in the double layer at the cold electrode, which causes the cold-side voltage and the overall Δ*V* to become stable and positive.

In summary, geopolymers reconstruct the gel framework and pore solution of i-TECs via a chemical reaction pathway that is distinct from that of OPC. The structural homogeneity of the N-A-S-H gel, the inherently higher ionic concentration, and the tunable alkali metal ion species together endow geopolymers with an i-TE potential that is far superior to that of OPC. However, the i-TE response of geopolymers is governed not only by the carrier concentration and transport pathways but also by multi-ion competitive transport in the pore solution and the electrochemical behavior at the electrode interfaces, all of which affect the macroscopic TE output. Furthermore, for practical applications, issues such as the high shrinkage, efflorescence tendency, and carbonation-induced performance degradation of geopolymers present engineering challenges that must be addressed. Therefore, how to overcome this intrinsic limitation and further boost the i-TE response through active approaches is the central issue addressed in the next section on modification strategies.

## i-TE Performance Optimization

Although cement slurry exhibits n-type behavior due to the thermal diffusion advantage of OH^−^, its extremely low *σ_i_* and high dependence on water environment make it difficult to directly apply in meeting the actual requirements of energy collection and sensing. Therefore, the exploration of strategies for modifying the i-TE properties of cement-based materials becomes the key to advancing i-TECs from mere phenomenological understanding to functional application. This section summarizes the current 3 types of strategies, including ion concentration regulation, ion conductive channel construction, and ion-electron (i-e) effect. The statistical results are shown in Table 1.

### Regulate ion concentration

In the pore solution of OPC, OH^−^ serves as the dominant charge carrier for thermal diffusion, as its high thermal migration rate. However, the coexistence and competition of cations such as Ca^2+^, K^+^, and Na^+^ during hydration partially counteract this advantage.

Introducing an external source of concentrated OH^−^ to suppress the relative contribution of cations has proven to be a simple and effective optimization strategy. Typically, researchers introduce external ions into cement-based materials via vacuum impregnation (Fig. 9). It has been demonstrated that after impregnating hardened cement paste in a 2 M NaOH solution under vacuum for 24 h, a substantial amount of external OH^−^ penetrates the pore network. This not only markedly raises the OH^−^ concentration but also inhibits the cation dissolution from the hydration products, thereby effectively reducing their competitive effect in thermal diffusion. As a result, the directional thermal diffusion of OH^−^ proceeds almost undisturbed. The *σ_i_* jumps from 0.012 S m^−1^ under sealed curing to 0.082 S m^−1^, and the *S_i_* stabilizes at −0.62 mV K^−1^ [48]. These findings further confirm the decisive role of a high OH^−^ concentration in strengthening the n-type i-TE response.

**Fig. 9.**
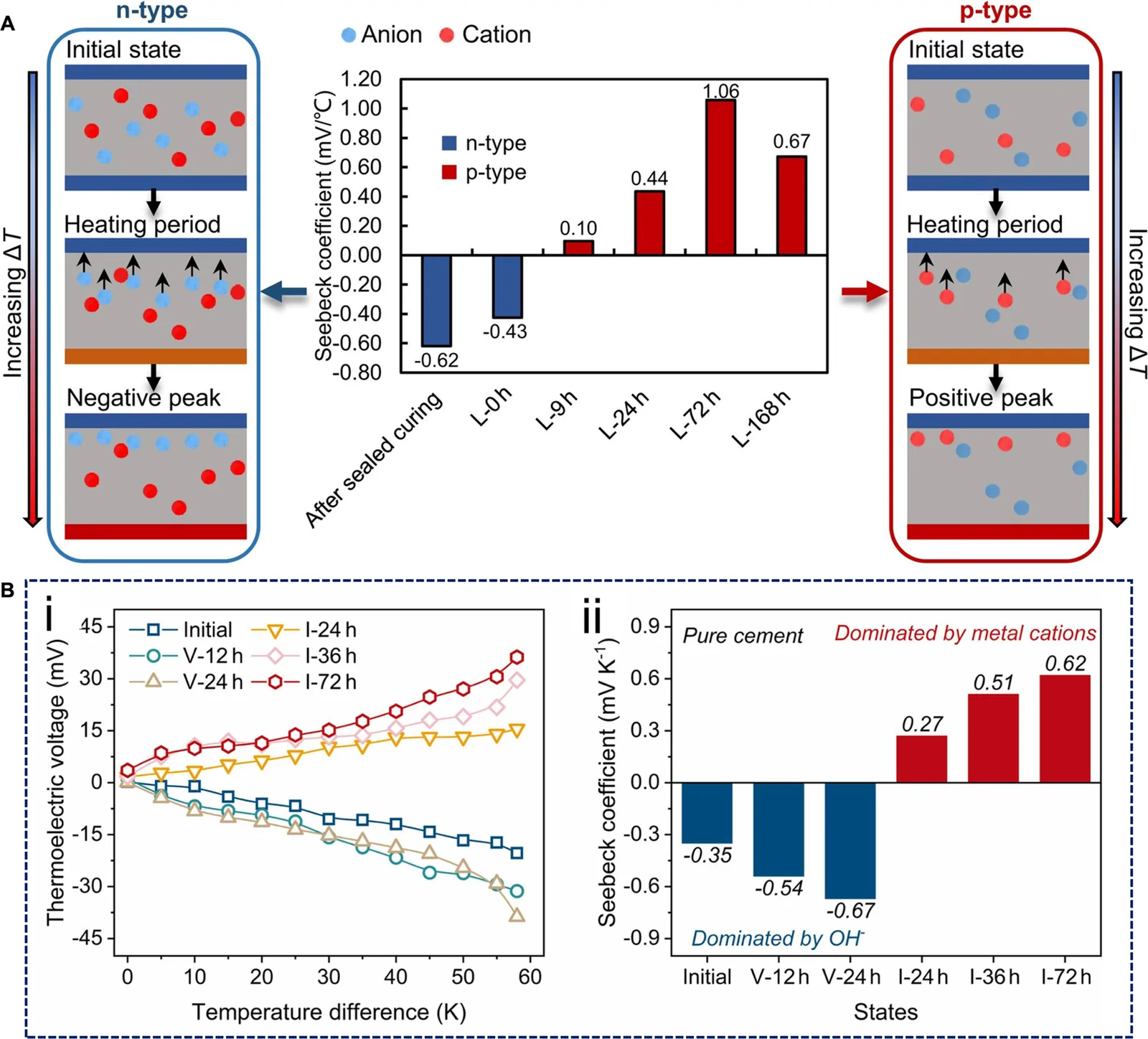
(A) *S_i_* of pure cement paste (L is leaching condition) and mechanism of i-TE effect. Reproduced with permission [48]. Copyright 2023, Elsevier. (B) (i) Thermal voltage response of pure cement under different states at varying temperature. (ii) *S_i_* of pure cement under different states. Reproduced with permission [93]. Copyright 2025, Elsevier.

Moreover, ion concentration control can not only reinforce the intrinsic n-type character of cement-based materials but also completely switch the material from n-type to p-type by altering the dominant ionic types. The underlying mechanism involves breaking the dominance of OH^−^ and rendering metal cations the primary charge carriers for thermal diffusion. One strategy is to selectively remove OH^−^. When hardened cement paste is immersed in deionized water, the concentration gradient drives the ions in the pore solution to diffuse outward. During the leaching process, due to the relatively high diffusion coefficient of OH^−^, it is the first to be lost, and the cation concentration in the pore solution rapidly increases. After leaching for 72 h, the *S_i_* of cement paste shifts from an initial −0.62 mV K^−1^ to +1.06 mV K^−1^ (Fig. 9A) [48]. A similar effect has been confirmed in KOH impregnated systems. As the immersion time increases, the *S_i_* changes from −0.35 mV K^−1^ to +0.62 mV K^−1^ [93], as shown in Fig. 9B. Furthermore, Lee et al. [133] employed acidic solution impregnation to efficiently eliminate OH^−^ via neutralization, achieving a p-type *S_i_* as high as +1.27 mV K^−1^. This result further demonstrates that the dominant carrier type in the system is highly dependent on the acid-based chemical equilibrium of the pore solution.

Another route to p-type conversion involves introducing high-valence cations into the pore solution to enhance the TE contribution from the cation side. For example, after impregnating a cement-based composite with a 0.01 M Ca(NO_3_)_2_ solution, the introduced Ca^2+^ compensates for the inherently weaker cation migration that occurs when OH^−^ dominates. This raises the TE voltage from 109.6 mV to 133.4 mV under ∆*T* = 45 K and yields a power factor of 0.62 μW m^−1^ K^−2^ [134].

The key advantage of the ion concentration tuning strategy is that it allows switching between n-type and p-type behavior merely by altering the ionic composition of the pore solution, and it can increase the *S_i_* by orders of magnitude within each regime. However, this strategy relies heavily on a sufficiently connected pore network and on the presence of water to sustain ion hydration and mobility. Its full effectiveness must therefore be matched with an optimized pore structure. Moreover, whether the introduction or leaching of foreign ions affects the long-term stability of cement hydration products, or even compromises the mechanical properties and durability of the matrix, requires more systematic investigation prior to engineering deployment.

### Constructing ion channels

Although regulating the ion concentration can reshape the type and quantity of dominant charge carriers, this approach remains constrained by the intrinsic pore structure of the cement matrix and cannot overcome the bottleneck that limits ion transport. In cement-based materials, gel pores and capillary pores are randomly distributed, tortuous, and locally isolated. It makes the actual ion transport path circuitous, high resistance, and low efficiency. Therefore, pre-introducing continuous, low-tortuosity, and highly selective transport pathways into the cement matrix has become an effective strategy for further enhancing the i-TE performance. To date, researchers have explored various strategies for constructing efficient ion channels, including biomimetic structural design, interfacial chemical anchoring, and interpenetrating double networks.

PVA hydrogel, whose molecular chains are rich in hydroxyl groups and can form a crosslinked network in water with excellent *σ_i_*, is regarded as an ideal candidate for constructing ion channels in cement matrix [135] (Fig. 10A). When PVA hydrogel is infiltrated into cement pores via vacuum impregnation, its hydroxyl groups form strong coordination bonds with Ca^2+^ in the hydration products. This not only strengthens the interfacial bonding but also provides relatively unobstructed migration pathways for OH^−^, thereby enabling selective ion transport within the channels. However, during dehydration-induced shrinkage, PVA hydrogel develops microcracks that disrupt the originally continuous ion channels, thereby impeding efficient ion transport. Experimental results show that the *S_i_* of cement with 15% PVA dropped from 28 mV K^−1^ to approximately 3 mV K^−1^, which may be linked to the microcracks generated by dehydration shrinkage of the PVA hydrogel [136] (Fig. 10B). To address the lack of channel continuity caused by random PVA infiltration, Wang et al. [54] drew inspiration from the directional transport structure of plant xylem and employed a bidirectional freeze-casting method to construct a directionally aligned cement skeleton. This skeleton was then infiltrated with PVA hydrogel to form a biomimetic multilayered ion channel (Fig. 10C). This structure selectively immobilizes Ca^2+^ while allowing OH^−^ to migrate freely, thereby further amplifying the mobility difference between anions and cations. The resulting composite achieves a high *S_i_* of −40.5 mV K^−1^, a *PF* of 168.6 μW m^−1^ K^−2^, and *ZT* of 6.6 × 10^−2^. At the same time, owing to the reinforcing effect of the aligned cement skeleton, the composite retains a respectable compressive strength of 15.1 MPa, thereby realizing an integration of i-TE performance and mechanical load-bearing function.

**Fig. 10.**
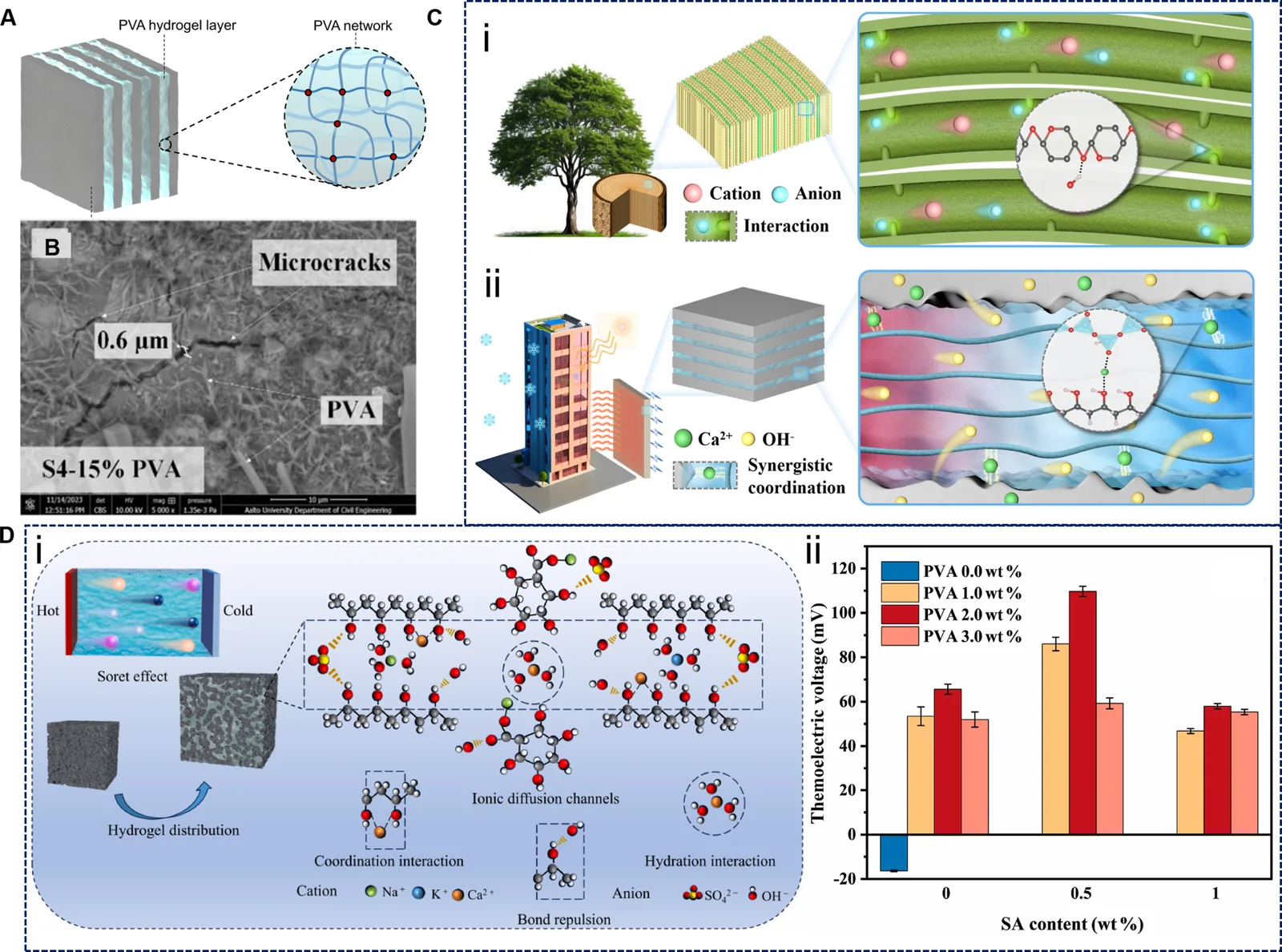
(A) PVA hydrogels were formed between the cement lamellae after freezing–thawing cycles [135]. Copyright 2023, Springer Nature. (B) Scanning electron microscopy (SEM) morphology microcracks formed due to PVA hydrogel shrinkage [136]. Copyright 2023, Elsevier. (C) (i) Schematic illustrations of the bioinspired design of CPC: The structure and selective ion transport in a wood stem. The microstructure of a plant stem can be simplified into fiber layers and xylem ray layers. The former contains lignin and cells, providing mechanical strength, while the latter contains vessels for transporting minerals and nutrients. (ii) Schematic illustrations of practical application, microstructure, and interfacial selective ion immobilization of CPC. Reproduced with permission [54]. Copyright 2025, Elsevier. (D) (i) Design principles for enhancing the i-TE performance of cement-based composites (PVA/SA hydrogel and ions within the cement-based composites with bonding interaction and hydration interaction). (ii) i-TE voltage at Δ*T* = 45 K. Reproduced with permission [134]. Copyright 2026, Elsevier.

The introduction of a PVA/sodium alginate (SA) double-network hydrogel offers greater flexibility in the chemical design of the ion channels. The 2 networks form an interpenetrating structure within the cement matrix. The PVA network stabilizes the channel skeleton via hydrogen bonding and coordination with the cement matrix, whereas the carboxyl and hydroxyl groups of SA introduce abundant negative charge sites onto the channel walls. This not only increases the Na^+^ concentration in the pore solution but also effectively suppresses the cross-channel migration of anions via electrostatic repulsion, thereby promoting the directional transport of cations [Fig. 10D(i)]. As shown in Fig. 10D(ii), when the PVA content is 2.0 wt % and the SA content is 0.5 wt %, the composite system achieves an *σ_i_* of approximately 0.071 S m^−1^ and a TE voltage of roughly 109 mV under ∆*T* = 45 K [134].

From the direct infiltration of PVA hydrogel to biomimetic aligned structures and further to double-network chemical crosslinking, it becomes clear that efficient i-TE channels require not only geometrically low-tortuosity pathways but also a matched interfacial chemical environment. It can ensure ion transport selectivity and structural stability under service conditions. However, ions cannot cross the electrode to enter the external circuit, resulting in the i-TECs system being confronted with the problem of charge accumulation and electrode polarization.

### i-e combination

In i-TE materials, ions cannot cross the electrode to enter the external circuit. Under a steady ∆*T*, they accumulate at the electrode–electrolyte interface and form an EDL. This capacitive operating mode implies that a purely ionic system cannot deliver sustained, usable electrical power. To overcome this limitation, researchers introduce an electronically conductive phase into the material, thereby exploiting the synergistic effect between ions and electrons. This coupling combines the high *S_i_* from ionic transport with the high electrical conductivity from electronic transport, enabling continuous charge delivery while preserving a large thermal voltage [137,138]. The i-e synergy operates in 3 stages [47] (Fig. 11A): (a) Cooperative migration. Under a temperature gradient, cations in the pore solution and holes (or electrons) in the functional filler migrate toward the cold side, thereby creating a potential difference. (b) Carrier compensation. Once the ∆*T* stabilizes, the ions accumulate at the electrode interface and build an internal electric field. This field then drives electronic carriers to drift in the opposite direction, counterbalancing the ionic potential until equilibrium is reached. (c) Steady output. After compensation, the stable thermal voltage arises solely from electronic carriers. However, owing to the optimized ion concentration and interfacial scattering effects, this steady voltage remains far higher than that of purely e-TE materials.

**Fig. 11.**
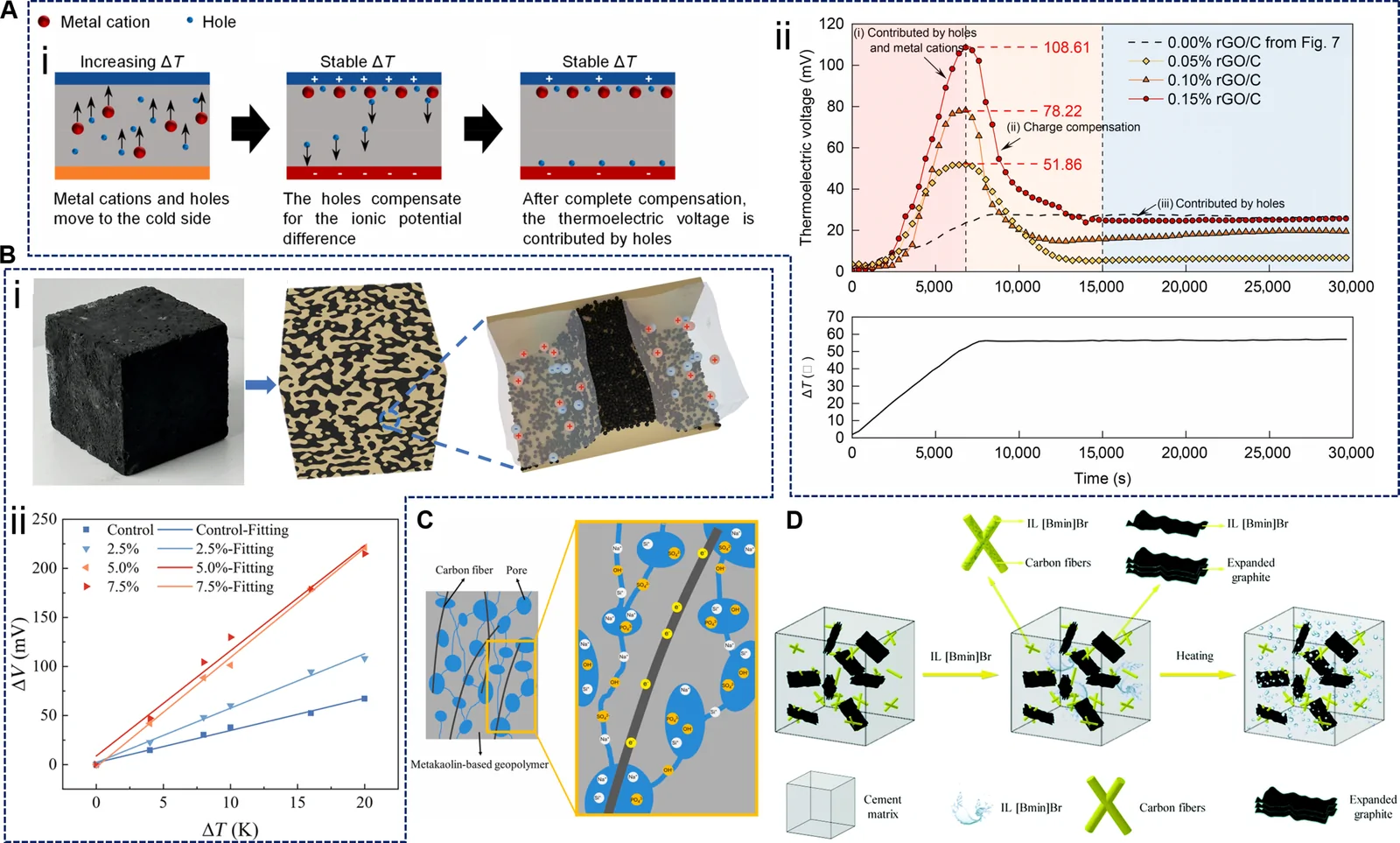
(A) (i) Cooperative working mechanism of i-e TECs. (ii) TE voltage evolution with temperature after leaching for 0.05% to 0.15% rGO/C. Variation of temperature difference versus time. Reproduced with permission [47]. Copyright 2022, Elsevier. (B) (i) Digital images and mechanism sketch of the internal electron and ion equilibrium of MPTEMs. The brownish-yellow structure represents the magnesium phosphate matrix (MPM) matrix, the sky-blue spheres with a negative sign represent anions, the pink spheres with a plus sign represent cations, the bright blue spheres represent electrons, the bright yellow spheres represent holes, the black spheres represent carbon black, and the transparent structure represents the pore solution. (ii) Fitting curves of the produced TE voltage difference corresponding to related temperature differences. Reproduced with permission [123]. Copyright 2025, Royal Society of Chemistry. (C) Comparison of ionic and electronic charge transport in porous geopolymer with embedded CF. Reproduced with permission [140]. Copyright 2026, Elsevier. (D) Schematic of the influence mechanism for the ILs [Bmim] Br on the Seebeck coefficient of expanded graphite and CF-reinforced cement composites. Reproduced with permission [142]. Copyright 2021, Royal Society of Chemistry.

Typically, carbon-based conductive fillers are incorporated into the cement matrix to build i-e transport network. Reduced graphene oxide (rGO) has attracted attention due to its high SSA, excellent hole conduction, and good interfacial bonding with the cement matrix. Studies indicate that in a 0.15% rGO-reinforced cement composite subjected to deionized water leaching, rGO provides hole-conducting pathways, while the dominant metal cations in the pore solution carry the ionic thermal diffusion [47]. Together, they raise the *S_i_* to 1,859.23 μV K^−1^ and the *PF* to 6.55 × 10^−2^ μW m^−1^ K^−2^, representing 4 orders of magnitude improvement over a purely electronic conduction system [47] (Fig. 11B). A similar effect has been demonstrated more systematically in MPC. When carbon black is added to the MPC matrix, its hydrophobic nature causes it to wrap around the pore solution, thereby forming individual i-TE micro-cells. These micro-cells are then interconnected in series and in parallel by the carbon black network (Fig. 11C). This composite enables a stable and sustained thermal voltage output under a single temperature-difference stimulus, achieving a maximum output exceeding 200 mV [123] (Fig. 11D). The i-e effect has also been observed in CF-reinforced geopolymer systems [139,140]. In these systems, CFs construct electronic conduction pathways and can connect isolated pores to form composite conductive channels (Fig. 11E).

Furthermore, ionic liquids, which are a class of organic salts composed of cations and anions and exist in a liquid state at room temperature, have recently been shown to further optimize i-e synergistic efficiency through interfacial engineering [141]. In EG–CF reinforced cement composite (EGCFRC), adding 3 wt % [Bmim]Br causes the *S* to jump from approximately −10 μV K^−1^ to −568 μV K^−1^, with a PF of 94.3 μW m^−1^ K^−2^ [142]. Although the authors did not explicitly discuss the i-e mechanism, the [Bmim]^+^ cations and Br^−^ anions migrate and rearrange under the temperature gradient, thereby creating high-density defects and energy barriers at the interfaces between the carbon materials and the cement matrix (Fig. 11F). These interfacial barriers scatter low-energy carriers while allowing high-energy carriers to pass, thereby raising the average carrier energy and markedly enhancing the *S_i_*.

The i-e strategy effectively enhances the i-TE performance of i-TECs by combining 3 benefits: improved *σ*, high thermal voltage from ionic transport, and optimized synergy through interfacial engineering. However, several key issues still hinder the engineering application of this strategy: the dispersion of carbon-based fillers, the long-term chemical stability of ionic liquids in the alkaline pore solution, and the performance degradation behavior of the mixed i-e system under service conditions.

## Applications

For building structures, the heat in their service environment comes mainly from solar radiation and geothermal heat. Although the resulting ∆*T* is on the order of 10 to 50 K, it covers a wide area and persists for a long time, which aligns with the advantages of i-TE materials in terms of high *S_i_* and low *κ*. As the performance of i-TECs continues to improve, their functional applications are expanding. Thus, research is advancing from fundamental materials studies toward device integration, with primary applications in energy harvesting, smart sensing, and self-energy storage.

### Building energy harvesting

By shaping i-TECs into building components, the building itself can be transformed into a low-grade heat collector. Typically, multiple p–n modules are connected in series to substantially increase the output voltage. However, the reported output voltages from cement- and geopolymer-based systems to date have generally remained insufficient for practical power applications [9]. Karalis et al. [143] fabricated 8 p–n pairs of geopolymer elements and connected them in series to form an i-TEG measuring 100 mm × 100 mm × 30 mm, as shown in Fig. 12A(i) and (ii). Under Δ*T* of 50 K, the device delivered a *V_oc_* of 28.5 mV and a maximum output power of 0.3 μW [Fig. 12A(iii) and (iv)]. Moreover, as presented in Fig. 12A(v), the output voltage and internal resistance remained stable over a continuous 5-h test, confirming the long-term operational reliability of i-TEC modules under steady ∆*T*. Moreover, when 9 specimens were connected in series and subjected to a ∆*T* of 150 K, the module generated an output voltage of approximately 2.3 V, which was sufficient to illuminate a light-emitting diode (LED) for about 4 s (Fig. 12B), demonstrating the capability to deliver sustained electrical power to a load [92].

**Fig. 12.**
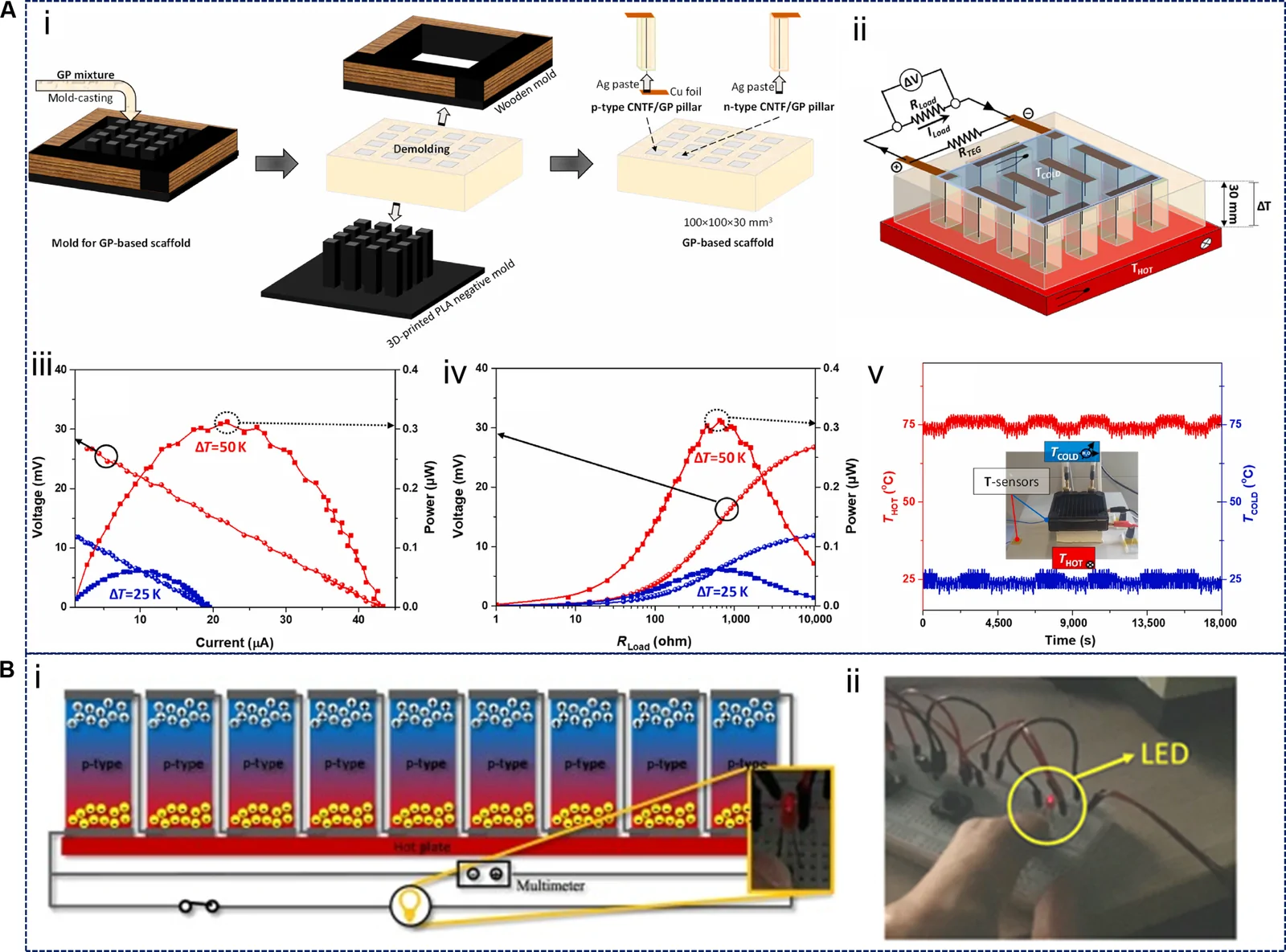
(A) (i) Schematics of the fabrication steps for the structural TEG assembly device. (ii) Corresponding equivalent electronic circuit. (iii and iv) Generated TE voltage, current, and power for dynamic loads at different applied Δ*Τ*. (v) Temperature profile stability monitoring for the structural TEG assembly device subjected to enforced through-thickness Δ*Τ* of 50 K for 5 h. Reproduced with permission [143]. Copyright 2024, Elsevier. (B) (i) TE energy harvesting for 9 cylindrical specimens in series and (ii) applied for LED light. Reproduced with permission [92]. Copyright 2025, Elsevier.

These advances illustrate that, through modular series–parallel design and capacitor integration, i-TEC devices can harvest the persistent low-grade ∆*T* present in building environments and thus provide support for self-powered infrastructure.

### Self-powered sensing systems

By utilizing the i-TECs itself as the sensing medium, self-powered structural damage warning and environmental perception can be integrated into buildings, relying solely on the TE voltage generated by internal ions in response to small temperature gradients [48,144]. Compared with electronic thermocouples, i-TEC sensors offer low cost, excellent compatibility with building structures, and the advantage of requiring no external power supply.

The i-TE effect of OPC was first validated for temperature sensing applications. Wei et al. [48] developed a cement-based temperature sensor by connecting 4 cement elements that had undergone vacuum saturation and 72-h deionized water leaching treatment in series [Fig. 13A(i)]. The sensor achieved a sensitivity of 3.98 mV K^−1^ under ∆*T* = 45 K, a value far exceeding that of traditional cement composites doped with CF or metal oxides. Its potential for building environmental monitoring and human thermal sensing was also demonstrated. When touched by a human hand, a Δ*T* of approximately 10 K triggered a fast voltage response of about 7 mV [Fig. 13A(ii)] [48]. Furthermore, the cyclic stability of the geopolymer system under Δ*T* was evaluated. The open-circuit voltage of the system did not decrease after multiple cycles, indicating its high stability and repeatability [124], as shown in Fig. 13B.

**Fig. 13.**
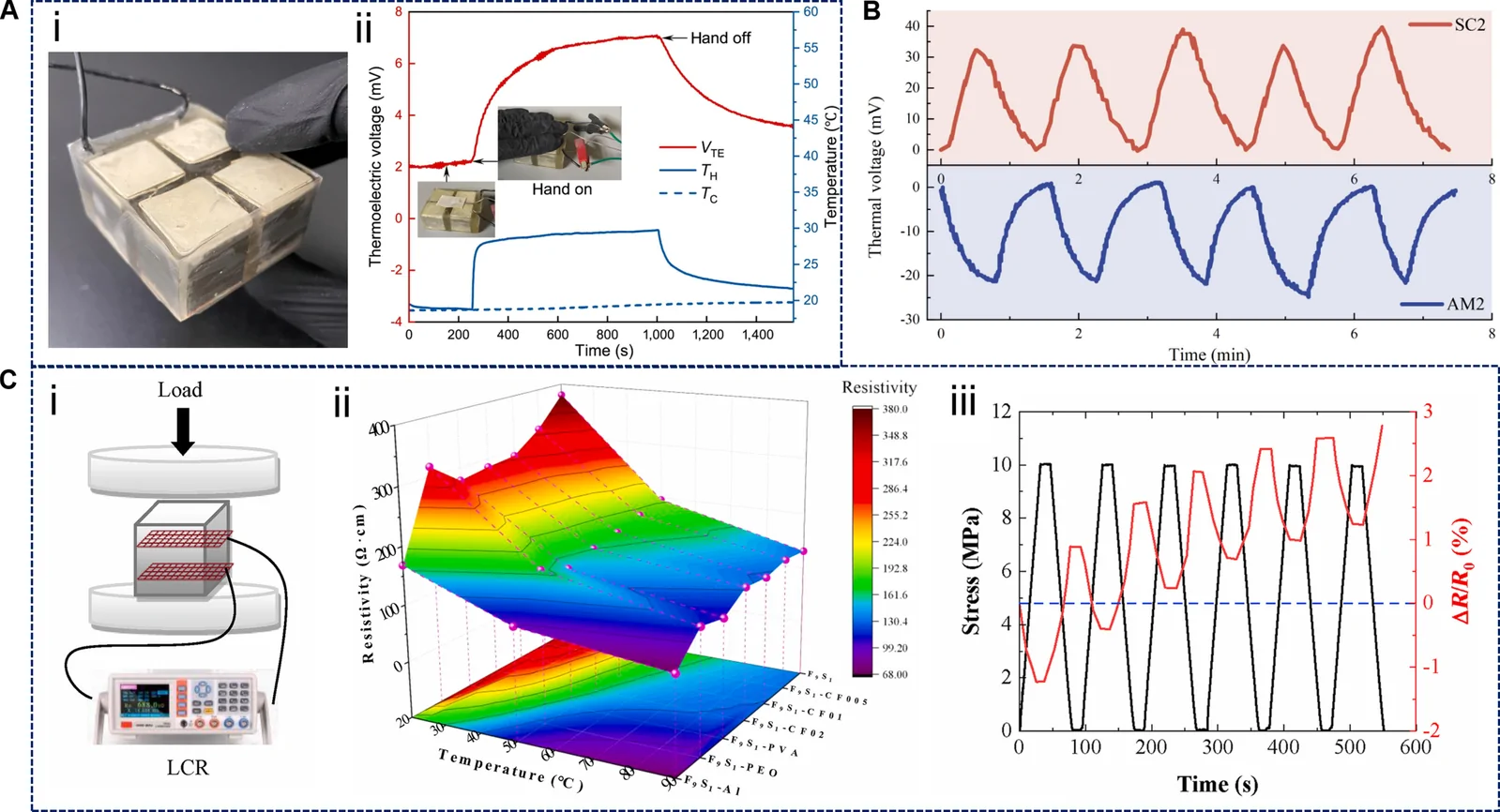
(A) (i) Cement-based sensors after sealing using epoxy resin. (ii) Temperature self-sensing ability of cement-based temperature sensor to sense human body temperature. Reproduced with permission [48]. Copyright 2023, Elsevier. (B) Curves of the TE voltage produced by the AM2 (blue line) and SC2 (red line) systems as a function of time for several alternating heating on/off cycles. Reproduced with permission [124]. Copyright 2026, Elsevier. (C) (i) Electrical property tests of AAM. (ii) Correlations between the resistivity and external temperature. (iii) Rate of change in electrical resistance of AAM submitted to cyclic compressive stress. Reproduced with permission [139]. Copyright 2025, Elsevier.

In addition, Qu et al. [139] investigated the sensing behavior of alkali-activated materials (AAMs) and found that their resistivity decreases substantially with increasing temperature, exhibiting a typical negative temperature coefficient effect and thereby demonstrating good temperature-sensing capability [Fig. 13C(i) and (ii)]. More importantly, under cyclic compressive loading, the fractional change in resistivity responded inversely to the stress level. Upon compression, the pores were squeezed and became more connected, thereby shortening the ion transport pathways and lowering the resistivity. Upon unloading, the pore structure recovered elastically, and the resistivity rose again [Fig. 13C(iii)]. This response mechanism differs fundamentally from that of conventional electronically conductive cement composites, where resistance changes arise from the modulation of tunneling current or contact resistance between conductive fillers. In the AAM system, the resistivity response is governed by changes in ion migration behavior under the combined action of stress and temperature fields [139].

From this, it can be seen that the sensing applications of i-TECs have expanded from single-parameter temperature detection to coupled thermo-mechanical sensing. Their core value lies in transforming the building envelope from a passive structural element that merely endures environmental actions into an active medium capable of perceiving environmental changes and internal damage. However, in both energy harvesting and self-powered sensing, a common practical challenge remains: The electrical energy generated by i-TECs under a ∆*T* will dissipate through capacitive discharge if not immediately utilized or stored. Therefore, simultaneously realizing energy conversion and electrochemical energy storage within the i-TECs will be key to overcoming this bottleneck.

### Electrochemical energy storage

Unlike conventional e-TE materials, i-TECs exhibit capacitive behavior. As ions cannot pass through the electrodes into the external circuit, the charges generated by i-TE materials under a temperature gradient accumulate at the electrode interface, forming EDLs. This feature allows them to serve directly as structural supercapacitors for simultaneous energy harvesting and on-site storage. As illustrated in Fig. 14A(i), during charging, the applied or thermally induced potential drives cations and anions in the pore channels to migrate toward and adsorb onto opposite electrodes, thereby forming EDLs of opposite polarity at the electrode–cement interface. During discharging, the adsorbed ions desorb and return to the pore solution, releasing the stored energy through the external circuit [145]. This process is highly analogous to the charging of a supercapacitor under an external electric field, with the only difference being that the former is driven by the temperature-gradient Soret effect rather than by an applied voltage.

**Fig. 14.**
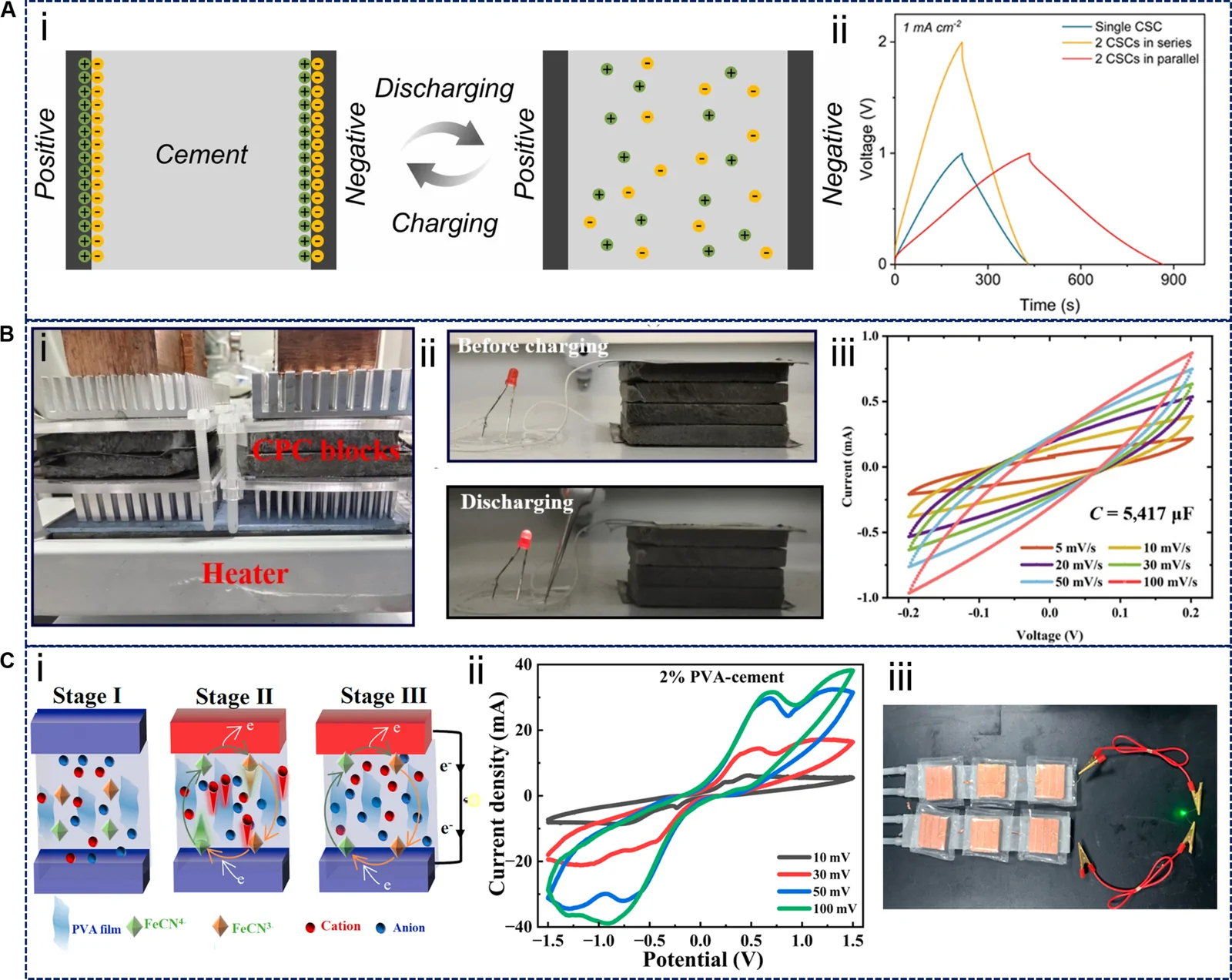
(A) (i) Schematic diagram of the energy storage mechanism in the cement-based supercapacitors (CSCs). (ii) CV curves of single CSC and 2 CSCs connected in parallel or in series. Reproduced with permission [93]. Copyright 2025, Elsevier. (B) (i) Photograph of the CPC module. The temperature difference between bottom side and top side of each pair was about 10 K. (ii) Photograph of a red LED powered by a wall model assembled by 4 CPC supercapacitors connected in series. (iii) CV curves with different scan rates of a CPC supercapacitor. Reproduced with permission [54]. Copyright 2025, Elsevier. (C) (i) Illustration of the TE mechanism of KFCN-PVA/cement. (ii) CV curves of the PVA2-KFCN. (iii) Glowing of green color LED bulb using the fabricated device. Reproduced with permission [146]. Copyright 2026, American Chemical Society.

Based on this principle, a symmetric solid-state cement-based supercapacitor was fabricated by introducing a foaming agent into OPC and assembling it with activated carbon electrodes [Fig. 14A(ii)]. It delivered an areal capacitance of 237.5 mF cm^−2^ at 0.5 mA cm^−2^ and an energy density of 32.9 μWh cm^−2^ [93]. Furthermore, 2 devices connected in series successfully powered a thermo-hygrometer. This work demonstrates that even without TE functional fillers, OPC possesses considerable electrochemical energy storage potential, owing to the ability of its pore-solution ions to form EDLs at electrode interfaces. Moreover, geopolymers exhibit even better storage performance than OPC due to their higher ion concentration and richer ionic species [139]. Wang et al. [54] integrate TE conversion and energy storage in a single biomimetic cement–PVA composite (CPC) module. The CPC material not only exhibited excellent TE performance but also possessed intrinsic energy storage capability owing to its multilayered structure. Four CPC supercapacitors connected in series successfully lit a red LED [Fig. 14B(i) and (ii)]. The cyclic voltammetry (CV) curves of a CPC block with carbon paper electrodes [Fig. 14B(iii)] showed a capacitance of 5,417 μF at a scan rate of 30 mV s^−1^, affirming the CPC’s suitability as a supercapacitor [54].

Building on this, coupling the Soret effect with the thermogalvanic effect provides a new strategy for overcoming the capacity limitations of purely EDL-based storage [Fig. 14C(i)]. By introducing the K_3_ [Fe(CN) _6_]/K_4_ [Fe(CN)_6_] (KFCN) redox couple into a PVA-based 3D ion channel network, the system maintains a high *S_i_* while simultaneously gaining a sustained electrochemical contribution from the thermogalvanic effect [146]. The redox reaction proceeds continuously, driven by the entropy difference between the hot and cold ends, thereby steadily supplying charge to the electrode interfaces. Unlike the pure cement system, the CV curve of PVA-KFCN shows distinct redox peaks at 0.25 and 0.45 V [Fig. 14C(ii)], with a peak current value of up to 40 mA, which is 4 times that of the pure cement system. The PVA-KFCN device operated stably for over 4,000 s under a ∆*T* of 10 K, and 6 devices connected in series successfully lit a small bulb [Fig. 14C(iii)].

From the initial validation of EDL capacitance in OPC, to the closed-loop demonstration of heat storage use in biomimetic structural modules, and further to the development of dual-mechanism coupled devices with long-term stability, i-TECs have shown great promise for integrating energy conversion and on-site storage into a single structural unit. In a building energy system, they can simultaneously function as both a TEG and a supercapacitor, eliminating the need for extra wiring, separate storage units, and complex power management modules. However, for practical engineering deployment, the energy density of EDL capacitance must still be substantially improved [147]. Furthermore, the long-term chemical compatibility and electrochemical stability of the electrode–cement interface require more systematic evaluation.

## Summary and Outlook

As an emerging material in the TE field, i-TE materials demonstrate great potential in converting ubiquitous low-grade thermal energy into electrical energy due to its high TE performance, low cost, and scalability [148]. Applying this concept to cement-based materials holds the promise of transforming vast building infrastructure into distributed energy harvesting and self-sensing networks. However, compared with mature polymer ionic gel systems, research on i-TECs is still in its infancy. Its progress toward practical applications remains hampered by inherent transport properties, interfacial constraints, and insufficient coordination between material design and device development. Consequently, this review outlines several promising yet underexplored research directions that are crucial for advancing i-TECs toward practical, sustainable, and multifunctional energy systems (Fig. 15):

**Fig. 15.**
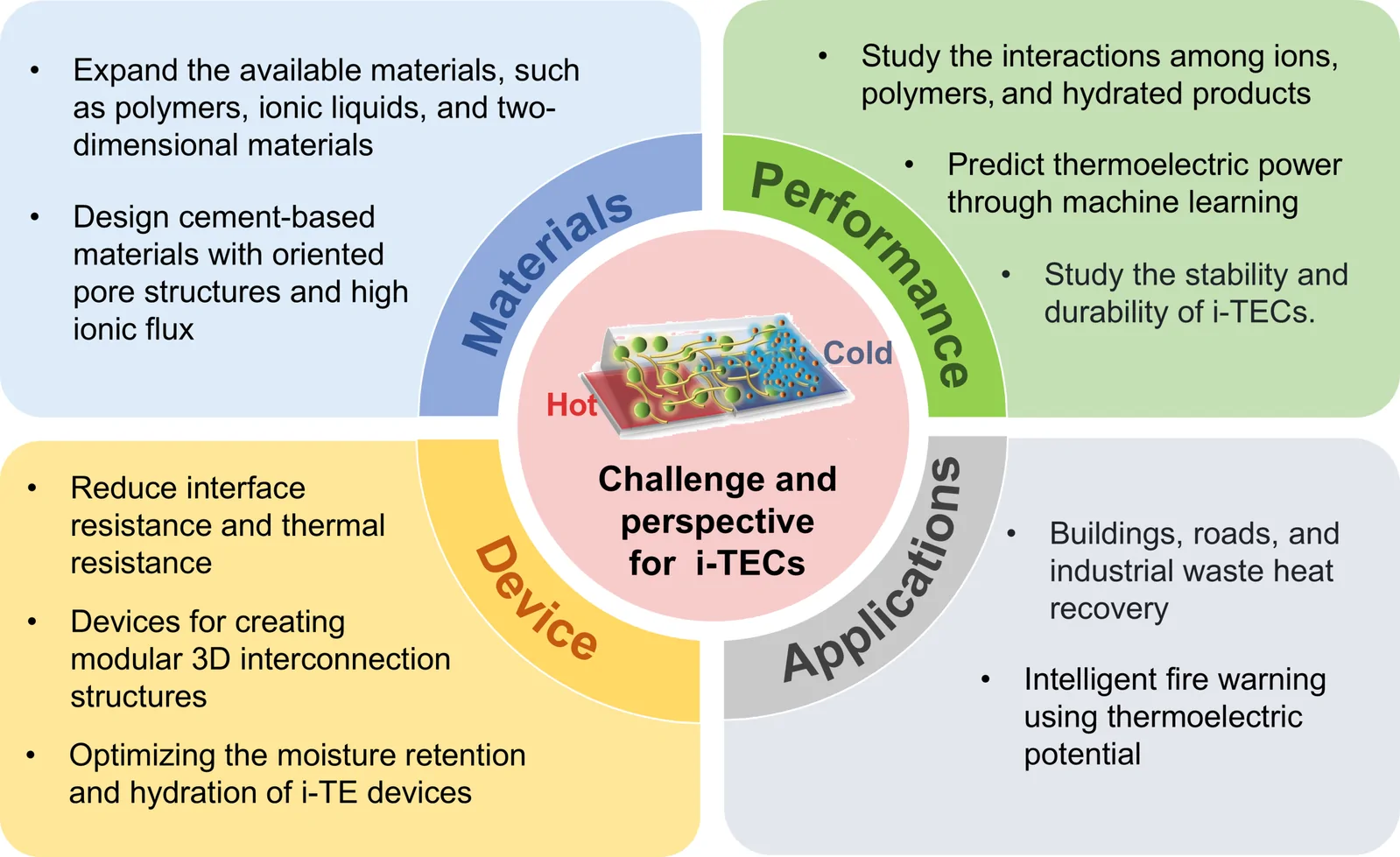
The challenges and prospects of i-TECs [163]. Copyright 2022, Springer Nature.

### Materials

The pore solution in cement-based materials is dominated by OH^−^, exhibiting n-type TE characteristics. Its *S_i_* is closely related to moisture content, pore structure (particularly mesopore proportion and connectivity), and ion concentration and type. Through effective regulation by strategies such as ion leaching or acid-base neutralization, a transition from n-type to p-type has been achieved. By utilizing PVA hydrogels to construct selective ion channels, or introducing carbon-based conductive fillers to build i-e synergistic transport networks, the *PF* and *ZT_i_* can be substantially enhanced. Concurrently, novel cementitious systems such as CSA cement, MPC, and geopolymers fundamentally optimize the ion types and transport environment, demonstrating superior i-TE potential compared to OPC.

In the future, further exploration could focus on introducing novel materials into cementitious materials that possess intrinsic ion conduction advantages or interfacial regulation capabilities. The field of polymer ionic gels has made remarkable progress in recent years, with core advantages including high *σ_i_*, remarkable toughness [149], and ionic selectivity that can be precisely controlled through molecular design [40,150]. Although attempts to composite cement-based materials with polymer hydrogels have shown initial potential, current compositing methods are still mainly physical filling and simple hydrogen bond coordination. Further research should systematically explore the interfacial bonding mechanisms at the molecular scale between ionic polymer and cement hydration products (C-S-H, CH, AFt/AFm phases), with a view to understanding the formation and evolution of the double layer at the interface. Based on this, organic–inorganic composite electrolytes with gradient compositions or interpenetrating network structures can be designed. Furthermore, the wide electrochemical windows, high *σ_i_*, and designability of ionic liquids (ILs) and deep eutectic solvents (DESs) also present opportunities for further research [141].

In addition, 2D materials such as graphene oxide (GO), MXene, and layered double hydroxides (LDHs) offer potential approaches for regulating the interfacial ion transport in i-TECs. Their layered structure, surface charges, and abundant surface functional groups can enhance their interactions with water molecules and ions. MXene has controllable surface termini, which can affect its surface charge, wettability, and ion–surface interactions [138]. At the same time, its high electronic conductivity may introduce ionic-electron coupling transport. The oxygen-containing functional groups on the GO surface can impart surface charge and hydrophilicity to the material. This feature makes it possible to regulate interfacial ion distribution, thereby promoting cation-selective transport in confined water environments [151]. LDHs are composed of positively charged layers and exchangeable interlayer anions, offering a potential mechanism for regulating anion distribution and migration [152]. Under appropriate assembly conditions, these 2D materials may form confined interfacial regions, in which surface charge effects and ion–surface interactions become more pronounced, thereby offering new possibilities for ion selectivity regulation [75]. However, it remains unclear whether such ordered ion transport and ion selectivity mechanisms can be maintained in cement-based systems. Cement pore solutions are characterized by high alkalinity, high ionic concentration, and continuous evolution, which may alter the dispersion state, interlayer spacing, surface chemistry, and ion selectivity of 2D materials. Therefore, the relationship between 2D material structure, interfacial ion distribution, and i-TE performance still requires further verification through direct experiments.

The pore structure of cementitious materials is a multiscale system ranging from nanogel pores to millimeter-scale capillary pores. However, current pore regulation strategies tend to focus on single-scale optimization, such as increasing the mesopore proportion to enhance SSA and EDL effect, or introducing millimeter-scale closed pores to reduce *κ*. Further design can draw inspiration from the hierarchical structure of efficient mass transfer systems in nature, constructing multiscale directional, low-tortuosity ion transport channels with macropore–mesopore–micropore synergy to fundamentally enhance *σ_i_*. In this framework, macropores (>100 nm) serve as the main trunks for ion transport, reducing overall transport resistance; mesopores (2 to 50 nm) provide high SSA, enhancing the EDL effect and interfacial ion enrichment, while micropores (<2 nm) enable selective sieving of specific ions through the confinement effect. By regulating the proportion and connectivity of pores at different scales, synergistic optimization of ion transport and i-TE potential can be achieved.

### Mechanism and performance

Current research has preliminarily clarified the i-TE mechanism dominated by the Soret effect in cement-based materials, identifying that the differential thermal migration of ions such as OH^−^, Ca^2+^, and K^+^ in the pore solution is the cause of i-TE potential. Meanwhile, the understanding of the i-e synergistic effect has evolved from simple performance superposition to the coupled physical process of internal electric field generation by ion migration under a temperature gradient, driving electron compensation and interface energy filtering.

Researchers could further establish a cross-scale theoretical framework. By coupling molecular dynamics (MD) simulations with macroscopic multiphysics transport equations, they could construct a cross-scale model capable of quantitatively describing the synergistic effect of complex ionic thermal diffusion and electronic conduction in cement porous media. It is also possible to study the interactions between ions and electrodes, ions and electrons, and ions and the matrix. Furthermore, the integration of computational simulation with artificial intelligence and big data technologies is expected to predict the performance of i-TECs, including mechanical properties, i-TE potential, and *ZT_i_*, to meet the requirements of various application scenarios.

However, the integration of i-TE functionality into cement-based materials inevitably introduces trade-offs with conventional cement properties that must be carefully managed for practical applications. The most fundamental trade-off is between mechanical strength and TE performance. High-performance i-TE modifications often require high porosity to enhance ion transport and EDL formation, which reduces compressive strength. PVA and SA hydrogels can enhance ion transport and water retention, but their long-term performance may be compromised by interactions with the highly alkaline cement pore solution. The bioinspired CPC retains 15.1-MPa strength, but this is still far below the strength level typically required in structural concrete applications [54]. Although the ion leaching method can regulate the TE response of cement-based materials, it will also reduce the overall ion concentration, which may affect the long-term alkaline environment required for steel passivation. Meanwhile, the i-TE functionality is highly sensitive to moisture, carbonation, and environmental degradation (see the Long-term stability and service life considerations section). Therefore, the enhancement of i-TE performance is often accompanied by the sacrifice of mechanical properties or the decline of long-term stability, and the comprehensive optimization of material performance remains a major challenge.

Future research should establish a multi-objective optimization framework that clarifies the weighting of various performance indicators for different application scenarios [153]. For structural applications (such as walls and columns), mechanical properties and durability are of utmost importance, while for nonstructural applications (such as paving and facades), TE properties may be more emphasized. The development of functionally graded materials represents a promising direction. It can maintain structural integrity while concentrating the i-TE functionality near surfaces.

### Interface and device-level integration

The transformation of i-TECs from small-size laboratory specimens to building TE modules capable of stable operation and large-scale deployment has undergone preliminary verification. The i-TEC modules have demonstrated the ability to power LEDs and charge capacitors driven by ∆*T*. Meanwhile, temperature sensors developed using their high *S_i_* have demonstrated sensitive responses to minute ∆*T*. These studies prove the feasibility of transforming building structures into distributed energy and self-sensing nodes.

In current experimental studies, the electrolyte–electrode interface has been widely identified as the main bottleneck limiting device performance. Slow charge transfer kinetics, persistent electrode polarization, and chemical and mechanical degradation of the interface during long-term service markedly limit the output voltage and long-term stability of i-TE performance [7]. However, there are obvious physical and chemical property mismatches between traditional metal electrodes (such as Cu, Ag, and Pt) and the cement matrix, and they are prone to corrosion or passivation in alkaline pore solutions. The mechanical bonding force at the interface between metal electrodes and cement-based materials is typically weak, making them highly susceptible to delamination under temperature cycling and humidity fluctuations. Researchers can develop embedded electrodes that are chemically compatible with the cement matrix and exhibit strong interfacial bonding and excellent electrical conductivity. It is noteworthy that carbon-based conductive materials and the cement matrix both belong to the inorganic carbon/silicate material system, with better matching thermal expansion coefficients, and the chemical stability of carbon materials in alkaline environments is far superior to that of ordinary metals. It is worth noting that carbon-based conductive materials and the cement matrix both belong to the inorganic carbon/silicate material system, with better matching thermal expansion coefficients. Carbon materials exhibit far greater chemical stability in alkaline environments than ordinary metals. Furthermore, the interfacial thermal resistance between the TE module and the environment is a key factor that cannot be ignored. Recently, Deng et al. [154] markedly reduced the interfacial thermal resistance and enhanced directional heat conduction by introducing copper-based thermal routing structure and thermal interface materials between the TEG and concrete. As a result, the open-circuit voltage increased by 61.2%.

Meanwhile, despite remarkable advances in laboratory-scale i-TEC device performance, a substantial gap remains between these achievements and practical building applications [155]. In terms of voltage output, individual i-TEC modules typically generate open-circuit voltages in the range of 10 to 100 mV under Δ*T* of 10 to 50 K [54]. While series connections of 4 to 8 modules can achieve 200 to 724 mV, practical low-power IoT devices typically require less than 3 V for operation [156]. This implies that 10 to 50 modules in series would be needed for a single sensor, presenting considerable integration challenges. For power density, i-TEC devices can achieve power densities ranging from 0.3 to 7.9 μW m^−2^ under laboratory conditions [146]. In comparison, typical low-power IoT sensors require less than 5 mW for continuous operation [156]. It means that even the most optimistic laboratory results, without considering losses, still require 633 m^2^ to be driven. Notably, voltage and power density alone are insufficient for a comprehensive assessment of practical feasibility. Matched-load power, internal resistance, energy conversion efficiency, and power management requirements must also be taken into account. In terms of sensing performance, i-TEC-based temperature sensors exhibit a certain level of sensitivity, but they still fall short of conventional thermocouples. This makes them viable for temperature sensing applications where power autonomy is prioritized over absolute accuracy.

When i-TE modules are scaled up to wall panels or pavement units, several factors degrade performance: (a) uneven temperature distribution across large areas, (b) variations in pore structure and moisture content, and (c) increased internal resistance from extended ion transport paths. Laboratory studies typically use small specimens under carefully controlled conditions, which are not representative of meter-scale building components.

Further research can develop standardized i-TEC module through 3D printing or multilayer assembly methods. Concurrently, high-performance hydrophobic packaging materials and thermal-moisture retention processes should be developed to ensure that i-TECs maintain sufficient internal humidity and the continuity of the ionic transmission channels. To bridge the laboratory-to-field gap, future research should focus on (a) developing large-area forming techniques (spraying, extrusion, formwork casting) suitable for in situ concrete processes, (b) establishing on-site quality control standards to ensure that laboratory performance can be replicated at engineering scale, (c) validating long-term reliability through field testing in real building environments, and (d) optimizing device architectures (module thickness, electrode configuration, series–parallel arrangements) for maximum output under realistic temperature gradients.

### Applications

Currently, preliminary explorations into energy harvesting, temperature sensing, and electrochemical energy storage have been carried out using i-TECs. However, the output voltage of individual i-TEC modules is typically only in the mV range, requiring large-scale series and parallel integration to achieve usable voltage and power levels. When TE modules are scaled up to wall panels or pavement units, their output performance deteriorates substantially due to uneven temperature distribution, pore structure variations, and electrode contact resistance. Therefore, it is necessary to develop large-area forming techniques (such as spraying, extrusion, or formwork casting) suitable for in situ or precast concrete processes, and to establish corresponding on-site quality control standards to ensure that laboratory performance can be replicated on an engineering scale. Furthermore, their long-term reliability and electrochemical compatibility have yet to be systematically validated through engineering testing.

Leveraging the advantages of i-TECs such as large *S_i_*, cost-effectiveness, and low emissions [157], large-area i-TEC modules could in the future be integrated into building envelopes, road surfaces, embankments, and other transport and municipal infrastructure. By generating electricity from absorbed solar thermal energy, it can power low-power IoT devices such as traffic flow sensors, structural strain monitors, and environmental quality detectors. Additionally, it can be used for fire early warning in building structures, integrating energy harvesting and sensing. For scenarios with high fire risk and where traditional monitoring methods are difficult to cover, such as tunnels, underground pipe galleries, and high-rise building curtain walls, the temperature difference-driven i-TE potential signal can respond within milliseconds, enabling real-time localization and graded early warning of the fire source location and intensity. Furthermore, the energy storage capabilities of i-TECs enable the structure to store the TE energy collected during the day in its own supercapacitors, releasing it at night or rainy days to ensure the all-weather continuous operation of the sensing nodes. In the future, the high reliability, long service life, and maintenance-free characteristics of i-TECs can be utilized to provide reliable energy solutions for monitoring and early warning systems in extreme environments.

## Data Availability

The data that support the findings of this study are available from the corresponding author upon reasonable request.
